# Environmentally-relevant exposure to diethylhexyl phthalate (DEHP) alters regulation of double-strand break formation and crossover designation leading to germline dysfunction in *Caenorhabditis elegans*

**DOI:** 10.1371/journal.pgen.1008529

**Published:** 2020-01-09

**Authors:** Luciann Cuenca, Nara Shin, Laura I. Lascarez-Lagunas, Marina Martinez-Garcia, Saravanapriah Nadarajan, Rajendiran Karthikraj, Kurunthachalam Kannan, Mónica P. Colaiácovo

**Affiliations:** 1 Department of Genetics, Blavatnik Institute, Harvard Medical School, Boston, Massachusetts, United States of America; 2 Wadsworth Center, New York State Department of Health, Empire State Plaza, Albany, New York, United States of America; 3 Department of Pediatrics, New York University School of Medicine, New York City, New York, United States of America; Department of Cell and Developmental Biology, Biocenter, University of Wurzburg, GERMANY

## Abstract

Exposure to diethylhexyl phthalate (DEHP), the most abundant plasticizer used in the production of polyvinyl-containing plastics, has been associated to adverse reproductive health outcomes in both males and females. While the effects of DEHP on reproductive health have been widely investigated, the molecular mechanisms by which exposure to environmentally-relevant levels of DEHP and its metabolites impact the female germline in the context of a multicellular organism have remained elusive. Using the *Caenorhabditis elegans* germline as a model for studying reprotoxicity, we show that exposure to environmentally-relevant levels of DEHP and its metabolites results in increased meiotic double-strand breaks (DSBs), altered DSB repair progression, activation of p53/CEP-1-dependent germ cell apoptosis, defects in chromosome remodeling at late prophase I, aberrant chromosome morphology in diakinesis oocytes, increased chromosome non-disjunction and defects during early embryogenesis. Exposure to DEHP results in a subset of nuclei held in a DSB permissive state in mid to late pachytene that exhibit defects in crossover (CO) designation/formation. In addition, these nuclei show reduced Polo-like kinase-1/2 (PLK-1/2)-dependent phosphorylation of SYP-4, a synaptonemal complex (SC) protein. Moreover, DEHP exposure leads to germline-specific change in the expression of *prmt-*5, which encodes for an arginine methyltransferase, and both increased SC length and altered CO designation levels on the X chromosome. Taken together, our data suggest a model by which impairment of a PLK-1/2-dependent negative feedback loop set in place to shut down meiotic DSBs, together with alterations in chromosome structure, contribute to the formation of an excess number of DSBs and altered CO designation levels, leading to genomic instability.

## Author summary

Faithful chromosome segregation during meiosis, the specialized cell division program that produces haploid gametes (i.e. eggs and sperm) from a diploid organism, is key for successful sexual reproduction. Diethylhexyl phthalate (DEHP), a commonly used plasticizer found in personal care and household products, has emerged as an endocrine disruptor that exerts reprotoxicity in mammals. In this study, we provide mechanistic insight into the modes of action by which environmentally-relevant levels of DEHP and its metabolites impair female meiosis in the *C*. *elegans* germline. Exposure to DEHP leads to defects in late prophase I chromosome remodeling, altered chromosome morphology in oocytes at diakinesis, errors in chromosome segregation, and impaired embryogenesis. Underlying these defects are higher levels of DSBs, altered DSB repair, defects in crossover (CO) designation/formation, germline-specific change in *prmt-5* gene expression and altered chromosome structure. We propose that DEHP exposure induces an excess number of DSBs by interfering with mechanisms set in place to turn off DSBs once CO designation is accomplished and by altering chromosome structure resulting in increased chromatin accessibility to the DSB machinery.

## Introduction

Errors in achieving accurate chromosome segregation during meiosis can result in the formation of either eggs or sperm carrying an incorrect number of chromosomes (aneuploidy) and has been implicated in 4% of still births, 50% of clinically-recognized miscarriages, congenital birth defects and infertility observed in humans [1–4]. Mounting evidence from mammalian studies suggests an association between exposures to environmental chemicals and aneuploidy [5]. However, thousands of man-made chemicals, including endocrine disrupting chemicals (EDCs) such as phthalates, are highly prevalent in the environment and their impact on meiosis is not fully understood.

Diethylhexyl phthalate (DEHP) is the most frequently used phthalate ester in the manufacturing of flexible polyvinyl chloride (PVC)-containing plastics, with approximately one to four million tons produced every year [6, 7]. Consequently, DEHP is present in a variety of consumer products including: clothing, personal care products, children’s toys, food packaging, vinyl flooring, carpets, wires, medical devices and construction materials [8]. The ubiquity of DEHP also extends to non-PVC materials such as makeup, adhesives, fillers, pills and printing inks [9]. Exposure to DEHP can occur *via* inhalation, ingestion and dermal absorption as it is detected in the air, water and soil, respectively [10, 11]. Once in the body, DEHP is rapidly metabolized by non-specific lipases and esterases in the gut, liver and blood into the primary monoester metabolite mono-(2-ethylhexyl) phthalate (MEHP), which is further oxidized to mono-(2-ethyl-5-oxohexyl) phthalate (MEOHP), mono-(2-ethyl-5-hydroxhexyl) phthalate (MEHHP), mono-(2-ethyl-5-carboxypentyl) phthalate (MECPP) and mono-[(2-carboxymethyl) hexyl] phthalate (MCMHP) metabolites excreted in the urine as glucuronides and sulfates [12–14]. The widespread use of DEHP underscores the importance of understanding how it impacts meiosis and thereby reproductive health.

DEHP and its metabolites are putative endocrine disruptors with the ability to interfere with both the male and female reproductive systems [15–18]. Male rats exposed to DEHP *in utero* exhibited altered gene expression in the fetal testes and altered androgenic steroidogenesis, resulting in decreased production of testosterone, hypospadias, cryptorchidism, shortened anogenital distance, reduced cell proliferation and increased apoptosis of Sertoli cells, and decreased sperm production and fertility [19–21]. In occupationally exposed Chinese men, elevated DEHP levels were associated with a significant reduction in serum free testosterone [22]. In female rodent studies, DEHP decreased 17β-estradiol levels, induced anovulation, disrupted estrous cyclicity and altered folliculogenesis in the ovaries [23, 24]. Furthermore, reduced primordial follicular reserve, oocyte quality and embryonic developmental competence were observed in the first three generations of female offspring from exposed female mice, indicating a DEHP-induced transgenerational effect [25]. Moreover, an association between DEHP exposure and endometriosis or decreased gestational age has been described in women [26]. However, while DEHP is a well-recognized agent of reproductive toxicity, the mechanisms by which it affects meiosis remain unclear. In addition, while studies conducted in rodents have mostly investigated the effects of exposures to high doses of DEHP (100–750 mg/kg bw/day), only a limited number have examined the effects from low dose exposures [23, 27, 28], which are more in the range of exposures detected in the general population. The latter, combined with the observation that DEHP displays a non-monotonic dose response profile, in which doses relevant to human exposure (0.5–25 μg/kg bw/day) led to reproductive toxicity in male mice, support the need of evaluating the effects of low dose exposure in the germline [27].

We used the nematode *C*. *elegans* to investigate the effects of exposure to low doses of DEHP in female meiosis. *C*. *elegans* is an ideal system for such studies given that meiosis in this system is well-characterized, this model organism has a rapid life cycle, and its cellular processes and pathways share a high degree of conservation with those found in mammals. Furthermore, *C*. *elegans* is a model that provides unique advantages for studies of reprotoxicity, including: (1) nuclei in the adult gonad are organized in a spatial-temporal gradient facilitating the study of meiosis and the clear visualization of alterations in chromosome morphogenesis at different meiotic stages resulting from the action of germline disruptors and (2) it is a highly predictive model of mammalian toxicities [29–32].

Here we identify the mechanisms by which internal circulating levels of DEHP and its metabolites, within the range detected in the general human population, exert reprotoxic effects in the *C*. *elegans* germline. We observed elevated levels of double-strand breaks (DSB), increased p53/CEP-1-dependent germ cell apoptosis, the presence of chromosomal defects in oocytes at diakinesis suggesting impaired DSB repair progression, and evidence of chromosome nondisjunction. DEHP also altered meiotic progression as shown by the presence of nuclei detected in late pachytene with markers of early prophase I, such as phosphorylated SUN-1 and DSB-1. These “laggers” also exhibited lower levels of COSA-1/CNTD1, indicative of lower levels of CO designation, and reduced Polo-like kinase-1/2 (PLK-1/2)-dependent SYP-4 phosphorylation (pSYP-4), suggesting a defect in the negative feedback loop mechanism normally set in place to turn off continued DSB formation in late pachytene. Finally, DEHP exposure resulted in germline-specific alteration in the expression of the arginine methyltransferase *prmt-5* gene as well as altered CO designation levels and increased X chromosome synaptonemal complex (SC) length in mid-pachytene nuclei. We propose that low dose exposure to DEHP alters meiotic progression, germline-specific gene expression, chromosome structure and negative feedback loop regulation of DSB formation, leading to excess DSB formation and altered CO designation.

## Results

### Environmentally-relevant levels of DEHP and its metabolites lead to X chromosome nondisjunction and altered meiotic progression

**Fig 1:**
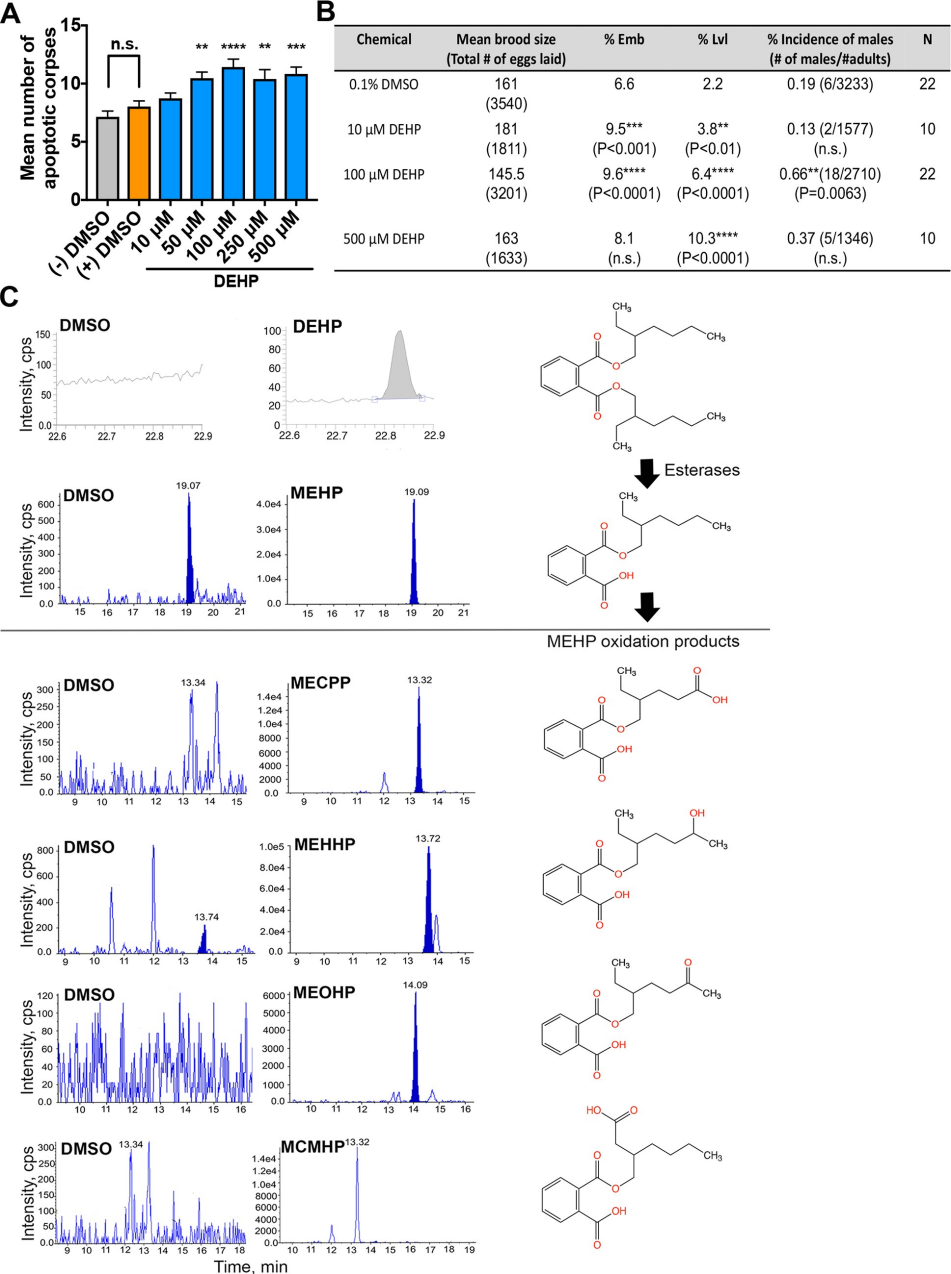
DEHP dose-response curve, plate phenotyping analysis and structures and representative GC-MS and HPLC-MS/MS chromatograms for DEHP and its metabolites. (**A**) Quantification of germ cell apoptosis, which takes place in late pachytene, from worms exposed to a range of DEHP concentrations compared to vehicle alone (0.1% DMSO). Analysis was done for three independent biological repeats, and more than 25 gonads were scored for each indicated exposure. Error bars represent SEM. **P<0.01, ***P<0.001 and ****P<0.0001 by the two-tailed Mann-Whitney test, 95% C.I. (**B**) The brood size, embryonic lethality, larval lethality and incidence of males were scored for the progeny laid by hermaphrodites continuously exposed to 10 μM, 100 μM and 500 μM DEHP starting from eggs until 24 hours post-L4 (adulthood) and compared to vehicle alone (0.1% DMSO). Indicated P values were calculated by χ^2^ analysis; n.s. = not significant. (**C**) Representative gas chromatography-mass spectrometry (GC-MS) and high performance liquid chromatography with tandem mass spectrometry (HPLC-MS/MS) chromatograms from the analysis of internal circulating levels of DEHP and its metabolites in worms exposed either to vehicle alone (DMSO) or to DEHP (left) and chemical structures of DEHP and its metabolites (right).

We recently identified DEHP in a high-throughput screen for environmental chemicals leading to increased X chromosome nondisjunction in the germline [33]. To further investigate how DEHP affects the germline, we first defined the environmentally-relevant (low dose) level of DEHP for our subsequent experiments. Given that DEHP exposure results in elevated levels of germ cell apoptosis in mice [34], we used germ cell apoptosis coupled with the assessment of numbers of eggs laid on plates and their viability, as readouts to identify the lowest dose at which DEHP induces a maximal apoptotic response accompanied by plate phenotypes suggestive of errors in chromosome segregation, without compromising the numbers of worms available for subsequent analysis. Worms carrying a *col-121(nx3)* mutation that increases their cuticle permeability to chemicals [35] were exposed from eggs to adults 24 hrs post-L4 (fourth and final stage in larval development) to various concentrations of DEHP (10 μM, 50 μM, 100 μM, 250 μM, and 500 μM). All concentrations between 50 μM to 500 μM elicited a significant increase in the levels of germ cell apoptosis compared to vehicle alone (0.1% DMSO) (P<0.01 as determined by the two-tailed Mann-Whitney test, 95% confidence interval (C.I.)) **(Fig 1A)**. Moreover, levels of germ cell apoptosis were not significantly different between (-) DMSO (untreated) and (+) DMSO control worms (P = 0.3343), suggesting that the toxicity exerted by DEHP is specific. We selected the dose of 100 μM DEHP for subsequent analyses because it resulted in a significant increase in embryonic lethality (Emb; P<0.0001 by χ^2^ test), which is suggestive of increased autosomal nondisjunction, and a significant increase in the incidence of X0 males (P = 0.0063), which is suggestive of errors in X chromosome segregation and corroborates the previous finding from our high-throughput screen [33, 36], without significant sterility (a reduction in the number of eggs laid) **(Fig 1B)**. Of note, the embryonic lethality and the incidence of males observed following exposure to 100 μM DEHP are not as elevated as in null mutants in which key events for achieving accurate meiotic chromosome segregation, such as chromosome synapsis or recombination, are abrogated [37, 38]. This may be due, at least in part, to inter-individual differences in the metabolism of DEHP, as previously observed in mice and humans [39].

To assess if 100 μM DEHP is an environmentally-relevant exposure dose, we determined the internal concentrations of DEHP and its metabolites in the worms by isotopic dilution mass spectrometric analysis of whole worm lysates [33]. Exposure to 100 μM DEHP resulted in internal median concentrations of 2.8 μg/g of DEHP, 2.6 μg/g of MEHP, 0.0305 μg/g of MEOHP, 1.2 μg/g of MEHHP, 0.0147 μg/g of MECCP, and 0.031 μg/g MCMHP (**Fig 1C**). These internal levels of the oxidative metabolites are within the range of previously reported circulating values detected both in the general female population in the U.S. and in pregnant women [40, 41].

**Fig 2:**
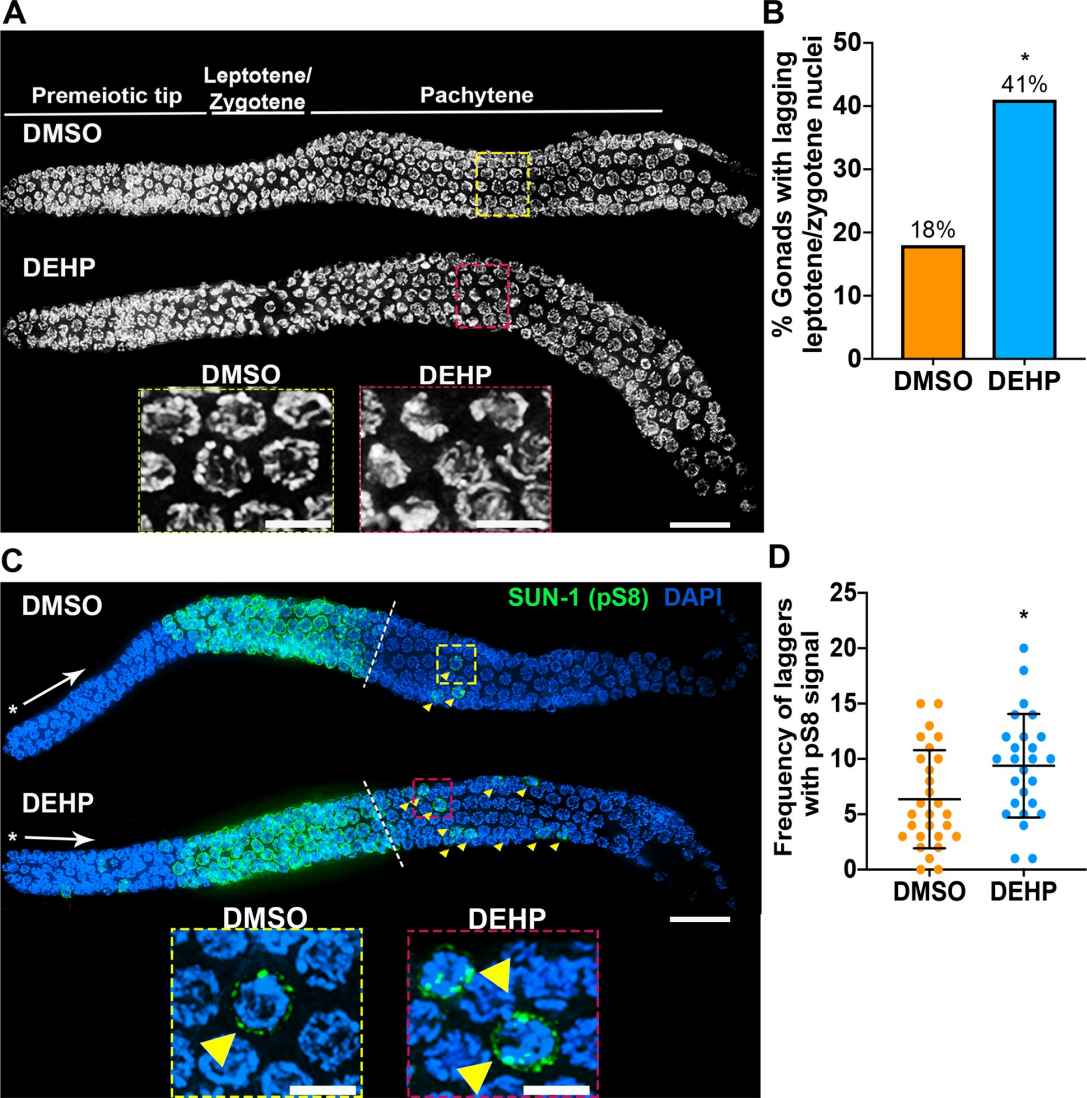
Meiotic progression is altered in the germline of DEHP-exposed hermaphrodites. (**A**) High-resolution images of whole mounted gonads from DMSO- or DEHP-exposed worms oriented from left to right. Insets show the presence of transition zone-like nuclei in mid-pachytene for DEHP-exposed gonads suggesting the presence of lagging leptotene/zygotene nuclei. (**B**) Quantification of gonads with lagging leptotene/zygotene nuclei. Data is from four independent biological repeats for DMSO (n = 39) and DEHP (n = 39; n = number of gonads scored, *P<0.05 by Fisher’s exact test). (**C**) Whole mounted gonads of chemically-exposed worms immunostained against SUN-1 (pS8) (green) show defects in meiotic progression as manifested by the persistence of pS8^+^ nuclei (“laggers”, indicated by yellow arrows) into mid to late pachytene. Asterisks indicate the premeiotic tip and white arrows show the direction in which nuclei move proximally in the germline. Hatched white lines demarcate the end of rows with all nuclei showing SUN-1 (pS8) signal (beginning of mid-pachytene). Insets show the presence of pS8^+^ lagging nuclei in the mid-pachytene regions of DMSO- and DEHP-exposed germlines. (**D**) Quantification of the number of pS8^+^ laggers observed in mid and late pachytene per gonad. Bars represent the mean number of laggers ± SD. *P<0.05 by the two-tailed Mann-Whitney test, 95% C.I.; Effect size = 5. Data was obtained from four biological repeats for DMSO (n = 28) and DEHP (n = 28); n = number of gonads scored. Scale bars, 5 μm.

Analysis of chromosome morphogenesis in adult germ cell nuclei from DEHP-exposed worms revealed lagging leptotene/zygotene nuclei in 41% (n = 39, where n = number of gonads scored, *P<0.05 by Fisher’s exact test) of the germlines, as shown by the presence of nuclei with chromosomes in a characteristic crescent-shaped organization among pachytene nuclei, compared to 18% (n = 39) for vehicle alone (0.1% DMSO) (**Fig 2A and 2B**). To determine whether meiotic progression might be affected, we examined the localization of phosphorylated SUN-1 (pS8), which forms bright aggregates at the nuclear envelope (NE) primarily during leptotene/zygotene and then becomes weaker and dispersed in the nucleus during early to mid-pachytene in wild type [42]. While most DEHP-exposed worms showed the timely appearance and disappearance of SUN-1 (pS8) signal (**Fig 2C and 2D**), a subset of worms displayed a statistically significant number of SUN-1 (pS8)-positive (+) nuclei extending into mid and late pachytene (P = 0.0115 as determined by the two-tailed Mann-Whitney test, 95% C.I.; Effect size = 5), suggesting problems with meiotic progression. The presence of pS8^+^ nuclei in the premeiotic tip of DEHP-exposed gonads (**Fig 2C**) is not suggestive of defects elicited by DEHP as pS8^+^ nuclei have been previously reported in the premeiotic tip of both wild-type and DMSO-treated worms as well as in normal mitotic nuclei in *C*. *elegans* embryos [33, 42–44]. Importantly, the presence of lagging leptotene/zygotene nuclei was not due to defects in the formation of the synaptonemal complex (SC) as evidenced by normal localization of SYP-1, a SC central region protein (**S1 Fig**) [37]. SC assembly was observed initiating in leptotene/zygotene, fully synapsed tracks were observed in pachytene nuclei, and the SC was fully disassembled by late diakinesis (-2 oocyte). The observation that only a subset of worms exhibits alterations in meiotic progression, and therefore that not all worms in a population are equally impacted by exposure to EDCs, is supported by our previous analysis showing that only 20–30% of worms exhibited defects in germline chromosome morphogenesis following dibutyl phthalate (DBP) exposure [33]. This variability is also evident in rodent studies in which control animals accidentally exposed to BPA *via* cage contamination showed striking variation in meiotic recombination among litters, as determined by quantifying the number of MLH1 foci [45]. Taken together, these data indicate that environmentally-relevant levels of DEHP can result in X chromosome nondisjunction and alter meiotic progression.

### DEHP alters chromosome morphology and remodeling in oocytes at diakinesis and leads to impaired early embryogenesis

**Fig 3:**
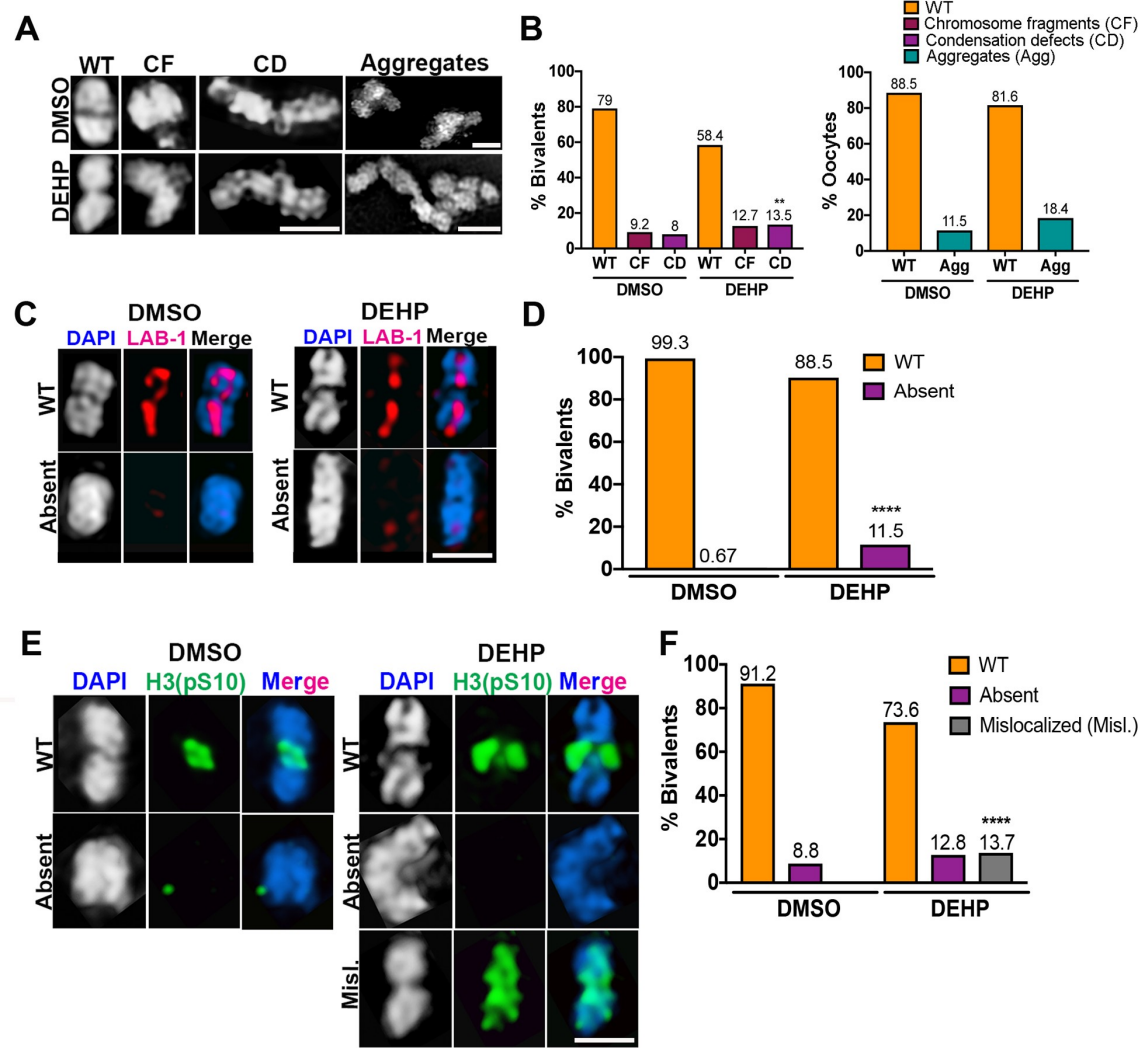
DEHP disrupts bivalent morphogenesis and chromosome remodeling. (**A**) High-resolution images of DAPI-stained bivalents in -1 oocytes at diakinesis exhibiting either normal morphology (WT) or defects including evidence of chromosome fragments (CF), elongated bivalents suggestive of condensation defects (CD), and chromosome aggregates (Agg) suggestive of end-to-end chromosome fusions. Note that DMSO also exhibits some degree of toxicity, as previously reported [29, 33, 96]. (**B**) (Left) Quantification of the % of bivalents displaying normal morphology or either chromosome fragments or condensation defects (n = 174 for DMSO from a total of 26 oocytes and n = 236 for DEHP from a total of 49 oocytes); n = number of bivalents scored. Numbers above each bar indicate percentages. (Right) Quantification of the % oocytes exhibiting chromosomal aggregates (n = 26 -1 oocytes for DMSO and 49 for DEHP). (**C, E)** Bivalents in the -1 oocyte displayed defects in chromosome remodeling as shown by (**C**) the absence of LAB-1 (n = 150 for DMSO and n = 227 for DEHP; n = number of bivalents scored) or (**E**) either the absence or mislocalization of phosphohistone H3 (pS10) (n = 137 for DMSO and n = 227 for DEHP). (**D, F**) Quantification of the % of bivalents displaying defects in LAB-1 and H3 (pS10) localization, respectively. Scoring of bivalents was done for two biological repeats. **P<0.01, ****P<0.0001 by Fisher’s exact test. Scale bars, 2 μm.

Analysis of -1 oocytes at diakinesis (the most proximal oocyte to the spermatheca) in DEHP-exposed worms revealed significantly elevated levels of aberrant chromosome condensation (13.5%, n = 236, where n = total number of bivalents scored, P<0.01 by Fisher’s exact test) compared to vehicle-control (8%, n = 174) (**Fig 3A and 3B**). Although we did not observe univalents (unattached homologs), it is possible that univalents were present in the chromosomal aggregates observed (**Fig 3A and 3B**). To further examine the extent of abnormalities observed in diakinesis nuclei, we assessed the localization of the Shugoshin functional homolog LAB-1, which functions as a regulator of sister chromatid cohesion (SCC) and localizes to the long arm of bivalents in diakinesis upon chromosome remodeling, and phosphorylated histone H3 (pS10), used as surrogate indicator of Aurora B kinase (AIR-2) activity, which localizes to the short arm of bivalents in -1 oocytes [46, 47]. We observed defects in chromosome remodeling as manifested by the absence of LAB-1 in 11.5% of bivalents (n = 227, P<0.0001 by Fisher’s exact test) in comparison to only 0.67% in DMSO (n = 150) (**Fig 3C and 3D**). H3 pS10 was no longer restricted to the short arm in 13.7% (n = 227, P<0.0001 by Fisher’s exact test) of DEHP-exposed bivalents relative to 0% (n = 137) in the DMSO vehicle control (**Fig 3E and 3F**). Furthermore, albeit not statistically significant, H3 pS10 signal was absent in 12.8% of DEHP-exposed bivalents (n = 227) relative to 8.8% in the DMSO vehicle control (n = 137).

**Fig 4:**
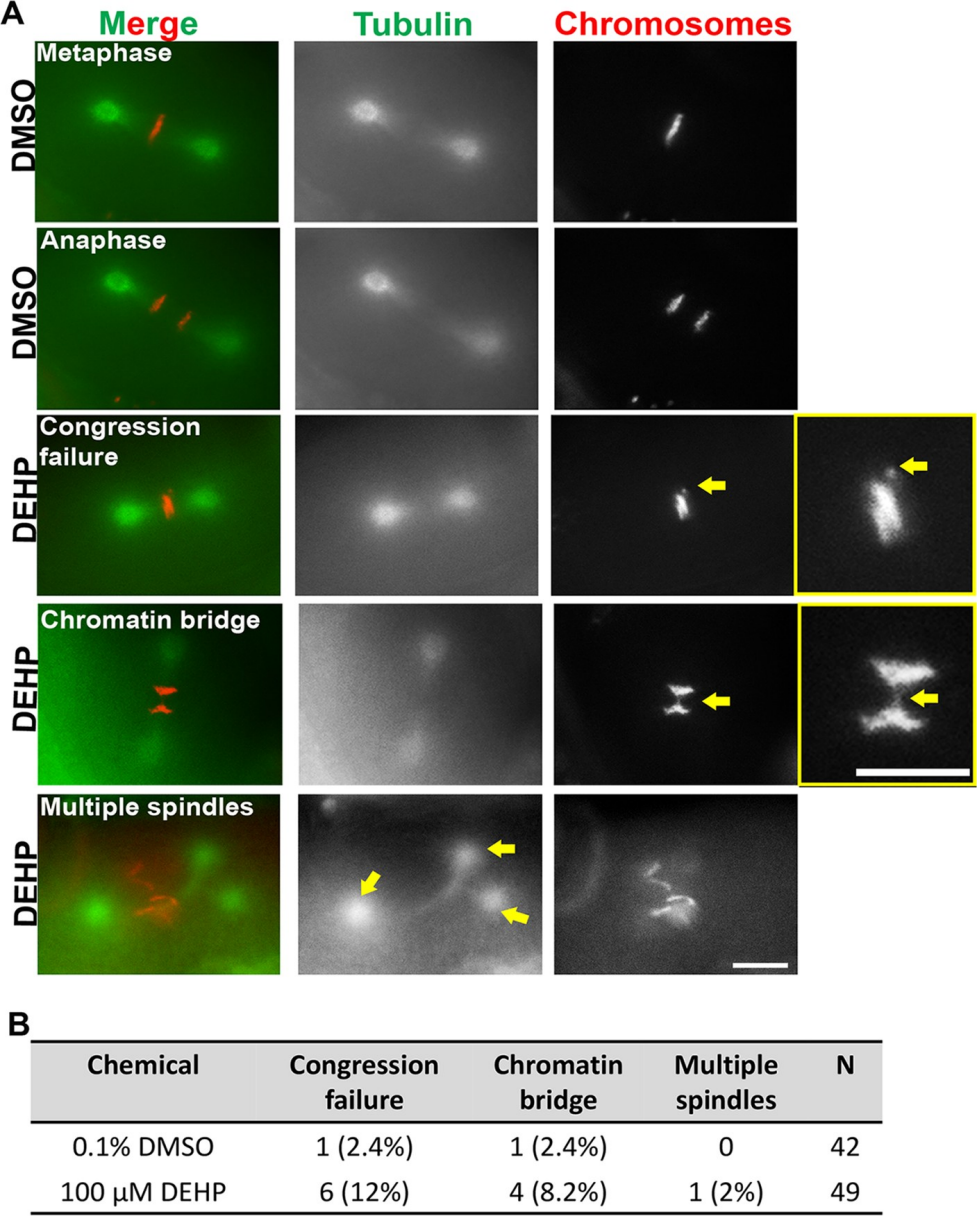
DEHP exposure causes defects in the first embryonic division. Representative images from time-lapse analysis of the first embryonic division in DMSO- and DEHP-exposed *H2B*::*mCherry; γ-tubulin*::*GFP; col-121(nx3)* worms. (**A**) DEHP exposure led to chromosomes failing to properly align on the metaphase plate (indicated by yellow arrow in inset), chromatin bridges at the metaphase to anaphase transition (indicated by yellow arrow in inset) and the formation of multiple spindles (indicated by yellow arrows). Chromosomes are shown in red and tubulin in green. Data for 0.1% DMSO (n = 42) and 100 μM DEHP (n = 49) was obtained from five biological replicates; n = total number of embryos scored from 42 and 49 independent animals, respectively. Scale bars, 5 μm. (**B**) Quantification of defects scored by time-lapse analysis of the first embryonic. A total of 7 out of 49 (14%) embryos displayed defects in the case of DEHP exposures, while only 2 out of 42 (4.8%) embryos showed defects in the case of DMSO. Four of the DEHP-exposed embryos exhibited more than one class of defect (see **S1 Table**).

To examine the effects of DEHP on early embryogenesis we performed live imaging analysis of the first embryonic division in chemically treated *H2B*::*mCherry; γ-tubulin*::*GFP; col-121(nx3)* transgenic worms. Pronuclear fusion, chromosome alignment at the metaphase plate and chromosome segregation at anaphase in the one-cell embryo was normal in DMSO-treated animals with only a small percentage (4.8%, n = 42; n = number of embryos scored) showing abnormalities. In contrast, congression failure, chromatin bridges at anaphase, and one instance of multiple spindles were observed in 14% (n = 7/49, where 4 embryos exhibited both congression failure and chromatin bridges) of one-cell embryos from DEHP-exposed worms (**Fig 4A and 4B** and **S1 Table**). Taken together, these data suggest that DEHP impairs chromosome morphology and remodeling in late prophase I as well as early embryogenesis.

### Germline apoptosis and double-strand break repair progression are altered in DEHP-exposed worms

**Fig 5:**
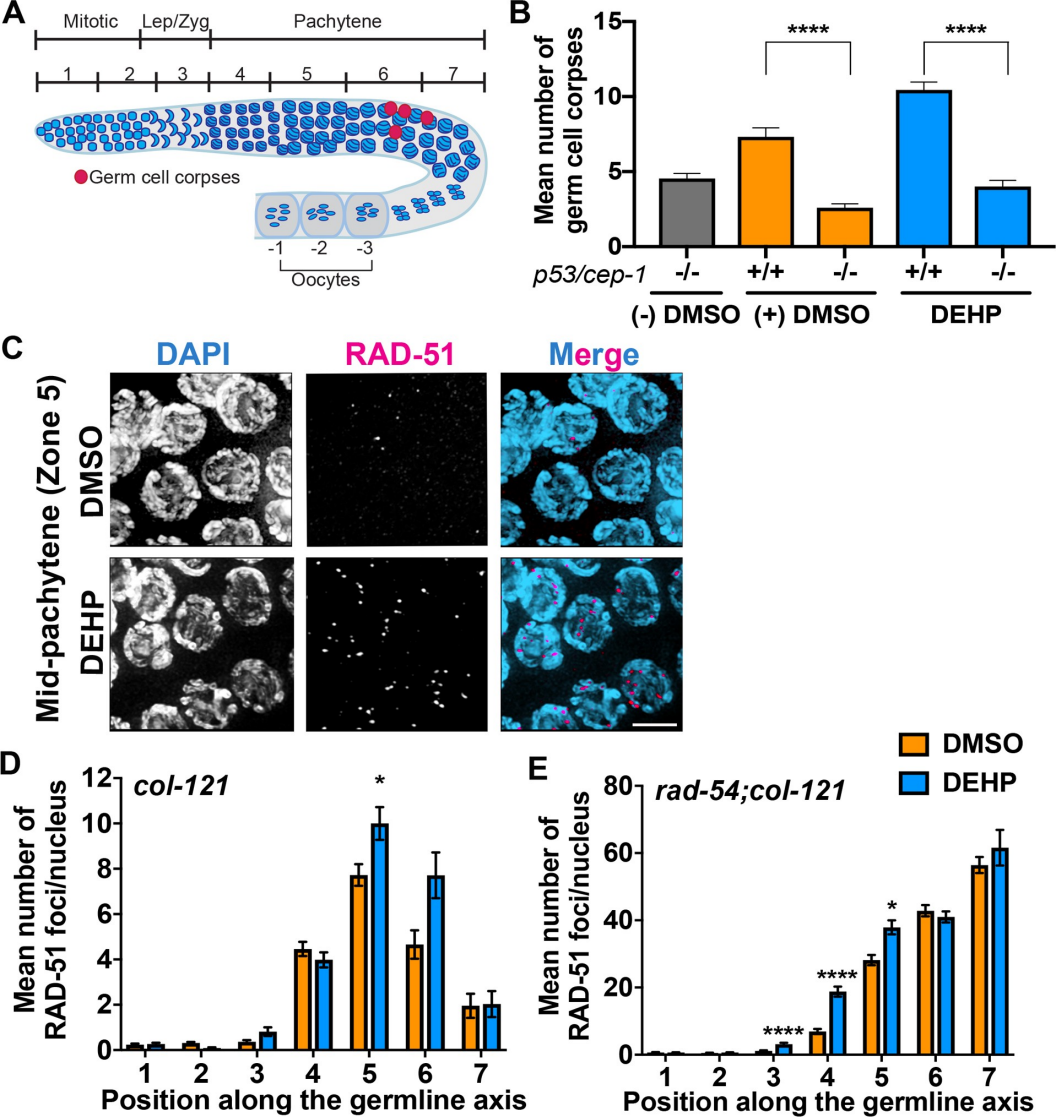
DEHP exposure leads to p53/CEP-1-dependent increased germ cell apoptosis, altered meiotic DSB repair progression and increased DSB formation. (**A**) Schematic of a *C*. *elegans* gonad depicting the seven zones for which RAD-51 was scored for all nuclei. Red circles represent nuclei undergoing apoptosis (germ cell corpses) which are detected close to the bend of the gonad arm at late pachytene. (**B**) Quantification of germ cell corpses in hermaphrodites exposed to 100 μM DEHP from embryogenesis until 24-hours post L4. 0.1% DMSO (vehicle control) and untreated ((-) DMSO) *cep-1(-/-)* worms were used as controls. Error bars represent SEM. Levels of germ cell corpses observed in *cep-1 (+/+)* worms were significantly higher than those observed in *cep-1 (-/-)* worms within each chemical treatment (****P<0.0001 by the two-tailed Mann-Whitney test, 95% C.I.). The levels of germ cell corpses observed in *cep-1 (+/+)* DEHP-exposed worms were significantly higher than those in the corresponding (+) DMSO vehicle control (****P<0.0001 by the two-tailed Mann-Whitney test, 95% C.I.). Between 27 to 47 gonads were examined for each chemical exposure from four biological replicates. (**C**) High-resolution images of mid-pachytene nuclei (zone 5) from whole mounted gonads co-stained with RAD-51 (magenta) and DAPI (blue). Scale bar, 3 μm. **(D)** Histogram shows the mean number of RAD-51 foci/nucleus (y-axis) scored along each zone in the germlines (x-axis) of *col-121* worms. Five gonads were scored *per* chemical treatment. A significant increase in levels of RAD-51 foci were observed for zone 5 of DEHP-exposed worms compared to DMSO. Error bars represent SEM from technical repeats for each of two biological replicates (*P<0.05 by the two-tailed Mann-Whitney test, 95% C.I.). (**E**) Histogram shows the mean number of RAD-51 foci/nucleus for each zone along the germlines of *rad-54; col-121* worms. Elevated levels of DBSs were observed beginning in early prophase I (zone 3) persisting until mid pachytene (zone 5). Error bars represent SEM for technical repeats from two to three biological repeats (*P<0.05, ****P<0.0001 by the two-tailed Mann-Whitney test, 95% C.I.).

Given the presence of chromosomal defects in oocytes at diakinesis (**Fig 3**) and elevated germ cell apoptosis in late pachytene (**Fig 1A**), we examined whether these might stem from defects in DSBR earlier during prophase I. DEHP-exposed worms exhibited p53/CEP-1-dependent increased germ cell apoptosis, as shown by the significant reduction in the levels of germ cell corpses observed in a *cep-1* homozygous mutant background, suggesting activation of a DNA damage checkpoint (P<0.0001 by the two-tailed Mann-Whitney test, 95% C.I.; **Fig 5A and 5B**). Since unrepaired DSBs and/or aberrant recombination intermediates persisting until late pachytene can result in activation of CEP-1-dependent germ cell apoptosis [48], we examined the progression of recombination by quantifying the levels of RAD-51 foci, a protein involved in strand invasion/exchange during DSBR [49], on immunostained whole-mounted gonads. In DMSO-treated worms, low levels of RAD-51 are observed upon entrance into meiosis, which then peak during early to mid-pachytene and decrease significantly by late pachytene as repair is completed. In contrast, a significant increase in RAD-51 foci was observed in mid-pachytene nuclei (zone 5; P<0.05 by the two-tailed Mann-Whitney test, 95% C.I.) in the germlines of DEHP-exposed worms (**Fig 5C and 5D**). To determine whether the observed increase in RAD-51 foci may be due in part to an increase in the number of DSBs we quantified the levels of RAD-51 in a *rad-54; col-121* mutant background. In the absence of the DNA repair protein RAD-54, which promotes RAD-51 displacement during strand-exchange, DSBR is blocked leading to accumulation and co-localization of DSBs and RAD-51 foci [50], hence, allowing for quantification of the total number of DSBs [51]. DEHP-exposed gonads showed a significant increase in RAD-51 foci in the leptotene/zygotene, early, and mid-pachytene stages (zones 3, 4, and 5, respectively; P<0.05) compared to vehicle alone (**Fig 5E**). Taken together, our data suggests that DEHP exposure results in elevated levels of DSB formation and altered DSBR likely resulting in the activation of p53/CEP-1-dependent germ cell apoptosis.

### DEHP exposure results in persistent DSB-1 signal in a subset of germline nuclei

**Fig 6:**
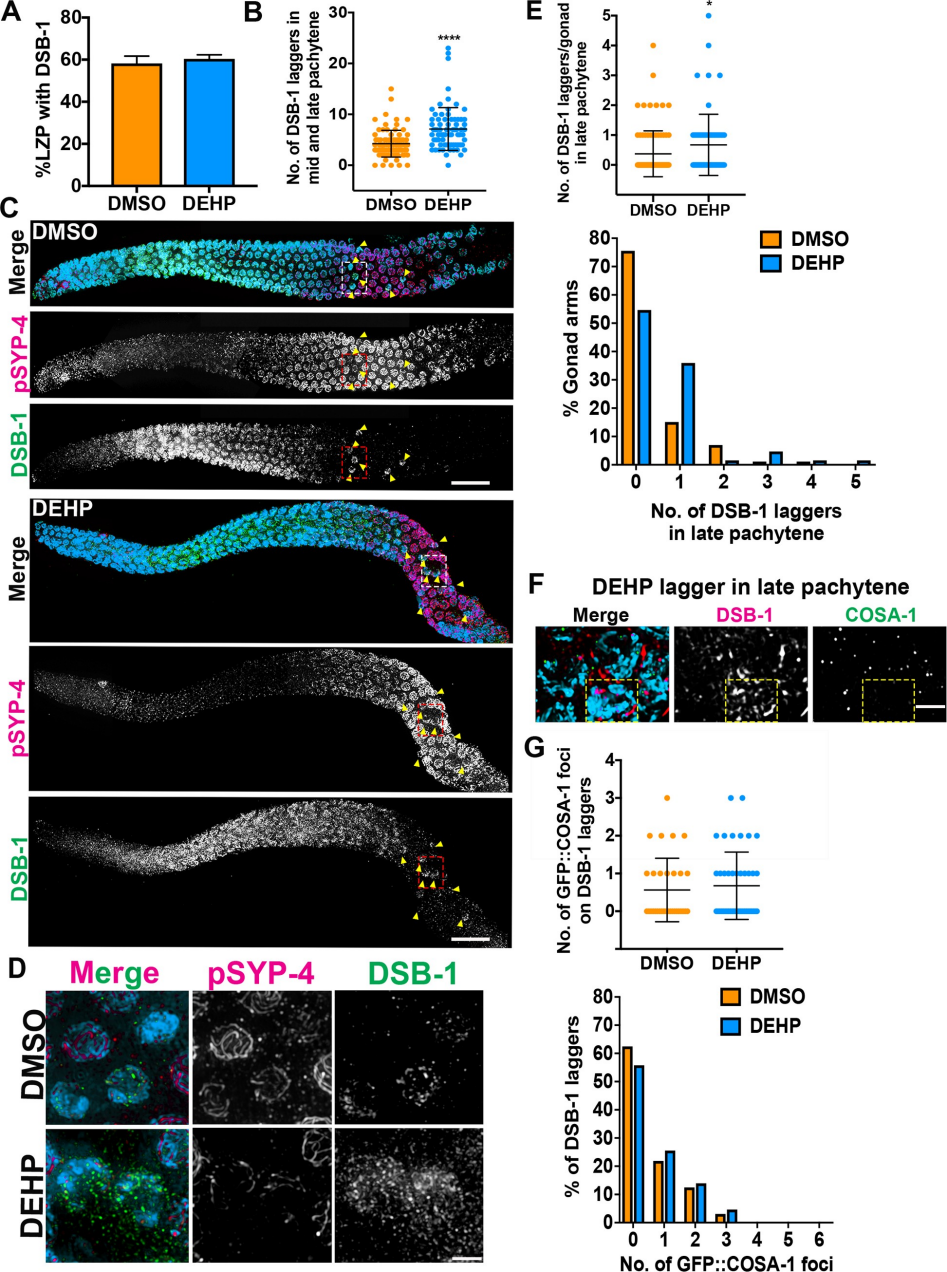
DEHP exposure results in DSB-1 laggers in mid and late pachytene and impaired CO designation. (**A**) Quantification showing that the % of the LZP (leptotene + zygotene + pachytene) region in the germline with rows where all nuclei show DSB-1 signal is not extended in DEHP-exposed worms compared to DMSO. **(B)** However, DEHP-exposed gonads showed elevated levels of DSB-1^+^ nuclei in the mid to late pachytene regions (DSB-1 laggers). ****P<0.0001 by the two-tailed Mann-Whitney test, 95% C.I. Data for DMSO (n = 85; n = number of gonads) and DEHP (n = 74) was obtained from four to eight biological repeats. Bars indicate mean ± SD. (**C**) High-resolution images of DAPI-stained gonads co-immunostained against DSB-1 (green) and phosphorylated SYP-4 (magenta) from DMSO- and DEHP-exposed worms. DSB-1 signal is observed in nuclei from leptotene/zygotene until early pachytene. DSB-1 laggers extend into mid and late pachytene (indicated by yellow arrows). pSYP-4 signal is detected in early pachytene and remains associated to chromosomes until late prophase I (given the presence of lagging leptotene/zygotene nuclei in pachytene resulting from DEHP exposure, note that early pachytene nuclei and the corresponding pSYP-4 signal are seen later in the DEHP-treated gonad compared to vehicle alone). Scale bars, 15 μm. (**D**) High magnification images of boxed DSB-1- and pSYP-4-stained mid-pachytene laggers in (C). DSB-1 laggers displayed reduced pSYP-4 signal compared to surrounding nuclei lacking DSB-1 staining. Scale bar, 2 μm. **(E)** (Top) Quantification of the number of DSB-1 laggers only in late pachytene from DMSO (n = 86)- and DEHP (n = 64)- exposed worms; n = number of gonads scored. Error bars represent mean ± SD. *P = 0.0138 by the two-tailed Mann-Whitney test, 95% C.I. (Bottom) Quantification of the % gonad arms displaying 0, 1, 2, 3, 4 and 5 DSB-1 laggers in late pachytene. **(F)** High magnification images of DSB-1 laggers from DEHP-exposed worms displaying less than six COSA-1 foci/nucleus. Scale bar, 2 μm. **(G)** (Top) Quantification of the number of GFP::COSA-1 foci scored for each DSB-1 lagger in late pachytene. (Bottom) Quantification of the % of DSB-1 laggers with 0, 1, 2, 3, 4, 5, and 6 GFP::COSA-1 foci. A reduction in the levels of GFP::COSA-1 is observed on DSB-1 laggers irrespective of chemical treatment.

In *C*. *elegans*, DSB-1 localizes to chromosomes during early prophase I (leptotene/zygotene), where it promotes the formation of programmed DSBs, and dissociates from chromosomes by mid-pachytene [52]. Because problems in early meiotic recombination events were previously shown to induce an extended DSB-1 zone [52], we quantified the region of DSB-1 localization by calculating the ratio of the length of the region encompassing DSB-1^+^ nuclei relative to the length of the gonad spanning the leptotene-zygotene-pachytene (LZP) stages [52]. Quantification of the DSB-1 localization region (expressed as % LZP) showed that DSB-1 progression is unaltered by chemical exposure (**Fig 6A**). However, we observed a significant increase in the frequency of DSB-1^+^ nuclei extending into mid and late pachytene for DEHP-exposed worms, referred to herein as “laggers”, compared to DMSO control (a mean of 7.1 laggers per gonad, n = 74, compared to 4.2 laggers, n = 85, respectively, where n = number of gonads scored; P<0.0001 by the two-tailed Mann-Whitney test, 95% C.I.; Effect size = 2.5; **Fig 6B and 6C**). In addition, based on the mean number of observed DSB-1 laggers (7.1) and the frequency distribution of DSB-1 laggers in DEHP-exposed worms (**S2 Fig**), 50% (n = 37/74) of DEHP-exposed worms showed ≥7 DSB-1 laggers in their germlines relative to 15.3% (n = 13/85) of DMSO-exposed worms. As in the case of SUN-1 (pS8) immunostained gonads, DSB-1 laggers displayed chromosomes in a crescent shape organization reminiscent of leptotene/zygotene stage nuclei (**Fig 6C and 6D**). These results suggest that DEHP exposure leads to a subset of nuclei persisting in a DSB-permissive state until late pachytene.

### Crossover designation is compromised in the germlines of DEHP-exposed worms

We previously showed that crossover (CO) designation is required for Polo kinase (PLK-1/2)-dependent phosphorylation of the structural component of the synaptonemal complex SYP-4 (pSYP-4), triggering a negative feedback mechanism that constrains the formation of further DSBs during meiosis [53]. Given the increase in DSB levels and the persistence of DSB-1 laggers detected in the mid to late pachytene regions of DEHP-exposed worms, we examined whether these may be linked to problems in CO designation and SYP-4 phosphorylation. Examination of gonads co-immunostained for DSB-1 and pSYP-4 revealed that DSB-1^+^ nuclei at mid-pachytene exhibited either reduced or no pSYP-4 signal throughout some chromosomes (**Fig 6D**). This is not due to downregulation of SYP-4 expression given that SC assembly, which requires normal expression of all SC components [54, 55], namely SYP-1, SYP-2, SYP-3 and SYP-4, is unperturbed in exposed worms (**S1 Fig**). Moreover, DSB-1^+^ nuclei at mid-pachytene also exhibited reduced PLK-2 signal (**S3 Fig**).

To examine whether defects with CO designation may account for the alterations in pSYP-4 signal in DSB-1 laggers, we quantified the levels of COSA-1 foci, a marker of CO designation, in chemically-exposed GFP::COSA-1 transgenic worms. GFP::COSA-1 foci were scored in both nuclei comprising the last six rows of late pachytene and in DSB-1 laggers in late pachytene (quantification was restricted to late pachytene, when six GFP::COSA-1 foci are clearly visible in wild type nuclei, thus ensuring even scoring among all chemical treatments). The majority of DSB-1-negative nuclei in late pachytene displayed a mean number of 5.9 COSA-1 foci (n = 448/485 for DMSO; n = 419/461 for DEHP, where n = number of nuclei scored; **S4A and S4B Fig**). However, the distribution of the number of late pachytene nuclei containing different levels of GFP::COSA-1 foci (between 0 to 10 foci per nucleus) was significantly different between the DMSO and DEHP chemical treatment groups (P = 0.0029 by χ^2^ test; **S4C Fig**), with more late pachytene nuclei from DEHP-exposed worms showing COSA-1 foci and higher numbers of GFP::COSA-1 foci compared to DMSO. While the number of DSB-1^+^ nuclei detected in late pachytene is low, DEHP-exposed worms showed a two-fold increase in the average number of DSB-1 laggers per gonad compared to DMSO (0.67 vs 0.37, respectively; DMSO, n = 64 and DEHP, n = 86; P = 0.0138 by a two-tailed Mann-Whitney test, 95% C.I.) (**Fig 6E**). Of note, regardless of chemical treatment, DSB-1 laggers in late pachytene showed defects in CO designation as evidenced by either a lack or a reduction in the number of GFP::COSA-1 foci detected in these nuclei (**Fig 6F and 6G**). Taken together, this analysis suggests that DEHP exposure: 1) leads to an increase in the number of late pachytene nuclei with altered CO designation levels, and 2) leads to an increase in the number of DSB-1 laggers in which CO designation and subsequent PLK-1/2-dependent phosphorylation of SYP-4 is either reduced or delayed.

### DEHP exposure leads to germline-specific change in the expression of the arginine methyltransferase *prmt-5*

**Fig 7:**
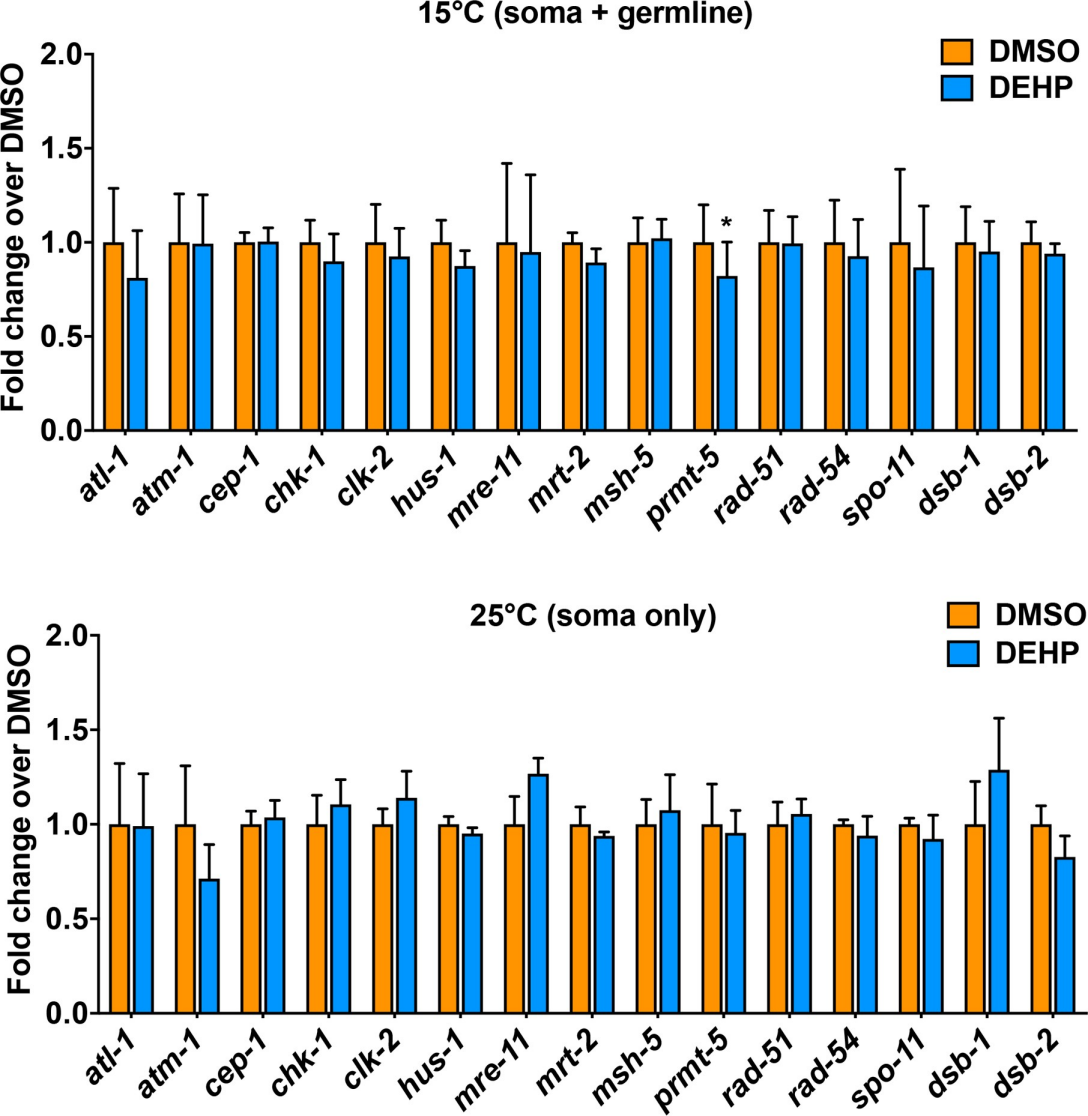
Germline-specific expression of *prmt-5* is altered upon DEHP exposure. Quantitative RT-PCR analysis in *glp-1(bn18); col-121(nx3)* worms, which grown at the permissive temperature (15°C) develop into worms with a germline compared to worms lacking a germline when grown at 25°C, revealed a significant germline-specific decrease in expression of *prmt-5* in DEHP-exposed worms compared to vehicle alone. Fold-enrichment over DMSO was determined using the ΔΔCt method. Experiments were done in three technical replicates from four biological replicates. Error bars indicate SEM. *P<0.05 by the unpaired two-tailed *t*-test.

As DEHP-exposed worms exhibited elevated levels of DSB formation and impaired DSB repair leading to increased p53/CEP-1-dependent apoptosis, we assessed germline-specific changes in the expression of 15 highly conserved genes involved in DSB formation, repair and DNA damage response (DDR) by qRT-PCR analysis. To this end, we used a *gpl-1; col-121* double mutant which forms a germline when maintained at 15°C, allowing for assessment of gene expression from both the germline and soma, but lacks a germline at the restrictive temperature of 25°C, providing a readout only for somatic gene expression. While DEHP exposure exhibited a weak tendency towards altered expression for some DSB formation, repair and DDR genes it resulted in significant germline-specific downregulation of only *prmt-5* (P<0.05 by a two-tailed unpaired *t*-test), an arginine methyltransferase involved in the modification of histones and receptors involved in signaling and transcription regulation, that could lead to altered gene expression or chromosome structure resulting in the observed increased levels of DSBs [56] (**Fig 7**).

### X chromosome SC length and CO designation levels are altered following DEHP exposure

**Fig 8:**
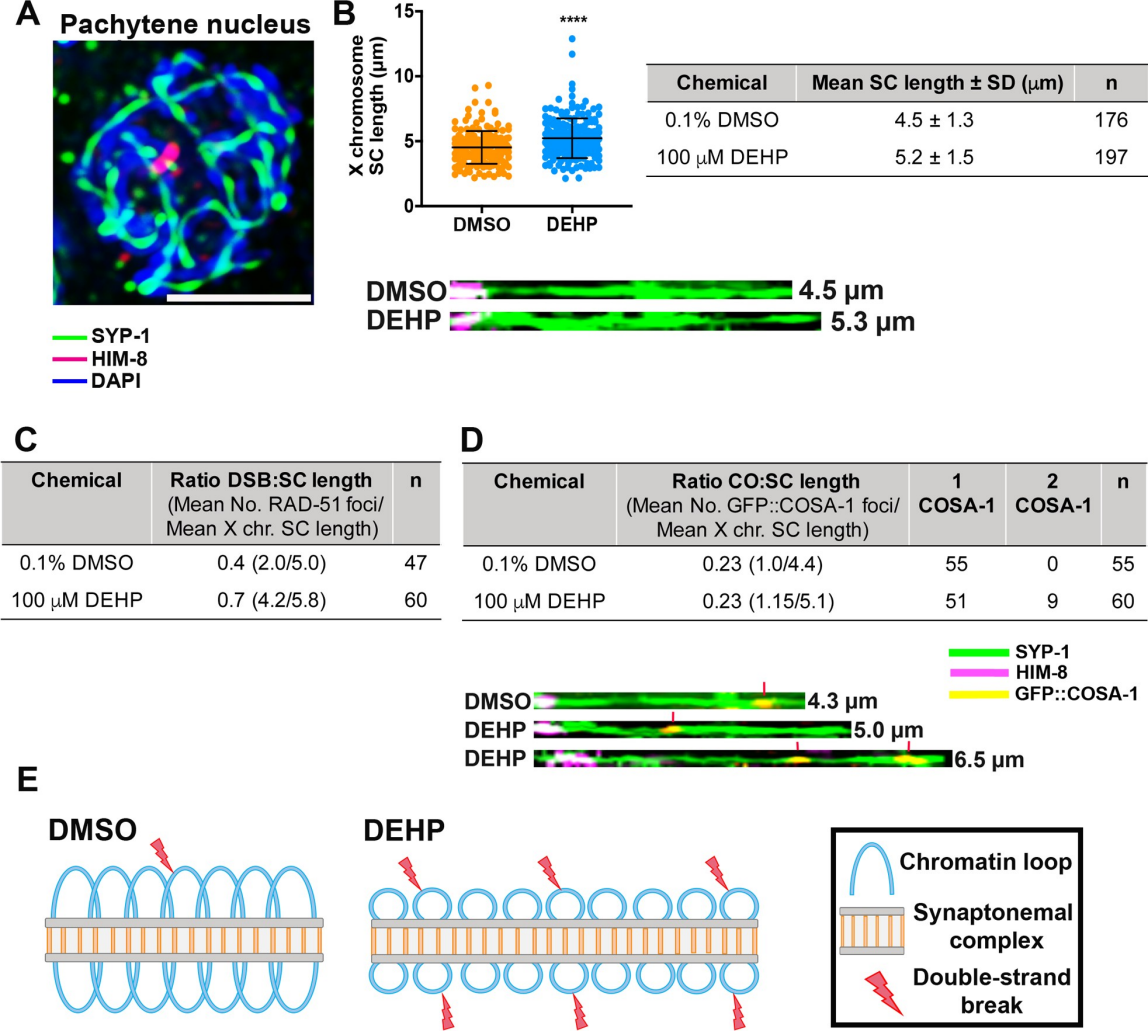
DEHP exposure alters X chromosome SC length and CO designation. **(A)** High magnification image of a full-projection of a mid-pachytene nucleus immunostained against the central region component of the SC, SYP-1 (green), and the pairing center end protein HIM-8 (magenta). Scale bar, 2 μm. (**B**) Scatter plot showing the distribution of X chromosome SC lengths in DMSO- and DEHP-exposed worms. The X chromosome SC length is significantly extended in DEHP-exposed pachytene nuclei compared to DMSO (****P < 0.0001 by the two-tailed Mann-Whitney test, 95% C.I.). Error bars represent mean ± SD. Shown on the right are the mean lengths of the X chromosome SC and the total numbers of nuclei scored (DEHP: n = 197 nuclei from 61 gonads, DMSO: n = 176 nuclei from 53 gonads; data collected from six independent biological repeats). Shown below the graph are X chromosomes, identified based on HIM-8 staining (magenta), traced in 3D along the SYP-1 signal (green) and straightened computationally using Priism 4.7. **(C)** Mean number of RAD-51 foci scored/mean length (μm) for computationally straightened X chromosomes from the same analysis. This analysis shows an increase in the ratio of DSB:SC length in pachytene nuclei from DEHP-treated worms. n = number of nuclei scored. **(D)** Mean number of GFP::COSA-1 foci scored/mean length (μm) for computationally straightened X chromosomes from the same analysis. Ratios of CO:SC length were the same for DEHP and DMSO treated worms. However, 2 COSA-1 foci per X chromosome were only detected in DEHP-treated worms and COSA-1 foci levels observed on the X chromosome were significantly higher in DEHP-treated worms compared to DMSO (*P<0.05 by a two-tailed Mann-Whitney test, 95% C.I.). Shown below are computationally straightened X chromosomes with HIM-8 (magenta), SYP-1 (green) and GFP::COSA-1 (yellow). Red vertical lines indicate COSA-1 foci. **(E)** Schematic illustrating the potential organization of chromatin loops in vehicle alone (DMSO) treated meiotic nuclei (left) compared to DEHP-treated nuclei (right). In the latter, the SC length is increased, which may be associated with decreased loop length and increased numbers of chromatin loops at which meiotic DSBs are proposed to take place.

In *C*. *elegans*, elongation of chromosome axis has been shown to alter the distribution and frequency of DSBs and COs along the chromosomes [51]. Given the elevated levels of DSBs and the altered expression of *prmt-5* observed in the germlines of DEHP-exposed worms, we investigated changes in chromosome structure by measuring the lengths of the SC along the X chromosome in mid-pachytene nuclei of DEHP-exposed gonads compared to control (0.1% DMSO). Furthermore, we assessed the levels of both RAD-51 and GFP::COSA-1 foci on the X chromosome of nuclei in the same region. The X chromosome was selected for analysis as it can be readily distinguished from autosomes by staining against HIM-8, which localizes at one end (pairing center end) of the X chromosomes promoting pairing interactions between the sex chromosomes [57]. The mean X chromosome SC length observed in pachytene nuclei from DEHP-exposed worms (5.2 ± 1.5 μm, n = 197) was significantly increased relative to DMSO (4.5 ± 1.3 μm, n = 176) (P < 0.0001 by the two-tailed Mann-Whitney test, 95% C.I.) (**Fig 8A and 8B**). Quantification of the number of RAD-51 foci detected on computationally straightened X chromosomes combined with analysis of the SC length revealed a mean number of 4.2 RAD-51 foci and a mean SC length of 5.8 μm per X chromosome on DEHP-treated worms (ratio = 0.7; n = 60 X chromosomes scored) compared to means of 2 RAD-51 foci and 5.0 μm SC length in DMSO (ratio = 0.4; n = 47 X chromosomes scored) supporting an increase in the number of DSBs relative to increased SC length in DEHP-treated worms **(Fig 8C)**. Quantification of the number of GFP::COSA-1 foci detected on computationally straightened X chromosomes revealed a significant increase in the number of GFP::COSA-1 foci in pachytene nuclei from DEHP-treated compared to DMSO-only exposed worms (P<0.05 by the two-tailed Mann-Whitney test, 95% C.I.). Means of 1.15 COSA-1 foci and of 5.1 μm for X chromosome SC length were observed in DEHP-treated worms (ratio = 0.23; n = 60 X chromosomes scored) compared to means of 1 COSA-1 focus and 4.4 μm X chromosome SC length in DMSO (ratio = 0.23; n = 55 X chromosomes scored) **(Fig 8D)**. Two COSA-1 foci were observed in 15% (n = 9/60) of the X chromosomes examined. 89% (n = 8/9) of the X chromosomes with 2 COSA-1 foci exhibited SC lengths higher than the mean for DMSO and 44.4% (n = 4/9) showed SC lengths higher than the mean for DEHP **(S1 Table)**. Given that in *C*. *elegans* COs are limited to one per homolog pair in most meiosis [58], these data indicate that DEHP exposure results in alterations (increases) in chromosome SC length that may be linked to the elevated levels of DSBs and increased CO designated sites observed following this chemical exposure (**Fig 8E**).

## Discussion

DEHP exposure is ubiquitous among populations throughout the world and it impairs a myriad of reproductive processes in both females and males. While previous studies have reported the effects of this phthalate on reproductive health, the mechanisms by which low dose exposure impairs the meiotic program have remained poorly understood. Here, we provide insight into the molecular mechanisms by which environmentally-relevant levels of DEHP impair meiosis in the context of a multicellular organism by using the *C*. *elegans* germline as a model. Our data suggests that DEHP exposure results in the alteration of early recombination events leading to defects in meiotic progression, chromosome remodeling in late prophase I and the alteration of chromosome structure and surveillance mechanisms operating at the level of DSB formation. Moreover, our study shows that *C*. *elegans* is a powerful tool for the study of phthalate-induced reprotoxicity.

### *C*. *elegans* provides a powerful system for understanding the effects on germline function of phthalates and their metabolites

An important goal of this study was to understand how environmentally-relevant levels of DEHP lead to germline dysfunction by impairing meiosis. Here, we found that the internal circulating levels of DEHP and its metabolites in the exposed worms are within the ranges reported in human biomonitoring studies: 9.9 μg/g for MEHP, 20.9 μg/g for MEOHP, 20.3 μg/g for MEHHP and 25.8 μg/g for MECPP in the urine of 100 pregnant Dutch women [40]. Meanwhile, in another study assessing the distribution of DEHP metabolites in follicular fluid and urine of infertile women in the United States, the authors reported median levels of 2.80 μg/g for MEHP, 0.02 μg/g for MEOHP and 0.15 μg/g for MEHHP [41]. Moreover, urinary concentrations of DEHP metabolites within the range reported for the U.S. general population were positively associated with increased pregnancy loss in subfertile women conceiving through medically-assisted reproduction [59]. Interestingly, we noticed that in the worm, unlike in humans, DEHP and MEHP were more abundant than the secondary oxidative metabolites, which may be due to species-specific differences in the metabolism of DEHP. Because worms lack a liver, where most of the DEHP metabolism occurs in mammals, metabolic processing of DEHP may be solely restricted to the esterases in the worm gut. Differences in the abundance of DEHP metabolic enzymes have been reported in mice, rats and non-human primates (marmosets), hence accounting for the differential levels of DEHP metabolites in these species [60]. Despite these species-specific differences in metabolism, our study provides evidence of reprotoxicity for environmentally-relevant levels of DEHP and its metabolites in the worm, as suggested by errors in X chromosome segregation detected in our HT screening of environmental germline disruptors and plate phenotyping analysis [33]. Importantly, our study highlights the suitability of *C*. *elegans* as a tool to evaluate the effects of phthalates on the germline as it displays conservation of the metabolic pathways mediating phthalate-induced toxicity in humans.

### DEHP exposure exerts germline toxicity through the impairment of a DSB formation surveillance mechanism involving PLK-1/2-dependent SYP-4 phosphorylation

In *C*. *elegans*, as in most other organisms, programmed meiotic DSB formation is initiated in early prophase I and chromosomes undergo multiple DSBs so that at least one is repaired as a CO (obligate CO) between each pair of homologs [61, 62]. Moreover, surveillance mechanisms are set in place such that DSB formation can be “turned off” after CO designation since continued DSB formation can be deleterious to the maintenance of genomic integrity [63, 64]. Here we show that exposure to environmentally-relevant levels of DEHP induces an excess number of DSBs and we suggest that, at least in part, this is linked to disruption of a negative feedback loop that inhibits DSB formation involving PLK-1/2-dependent SYP-4 phosphorylation and CO designation. Our data suggests that defects in CO designation/precursor formation in a subset of DSB-1-stained laggers activates a genome-wide response that precludes their exit from a prolonged DSB-permissive state. This is further supported by a previous study showing that defects in CO formation on one or more chromosomes triggers a DSB-1-mediated DSB-permissive state in mutants with defects in early recombination steps [52]. Several studies have proposed that nuclei maintaining a DSB-permissive state have an extended window for DSB formation to assure proper CO designation/precursor formation on all chromosome pairs [52, 53]. Here, our RAD-51 foci analysis in a *rad-54* mutant background revealed a greater number of DSBs introduced throughout the early stages of prophase I upon DEHP exposure. In a subset of nuclei, this was coupled with an absence of the pro-CO factor COSA-1 on some or all chromosomes and either a reduced or absent signal of phosphorylated SYP-4, respectively. We have previously shown that chromosomes failing to achieve CO designation are devoid of pSYP-4 and that PLK-1/2-dependent phosphorylation of SYP-4 may serve as a mechanism to shut down DSB formation upon CO designation [53]. Therefore, our current analysis suggests that DEHP impairs meiosis in part by disrupting the negative feedback loop between CO designation/precursor formation and SYP-4 phosphorylation, leading to a deregulation of DSB formation on chromosomes lacking an “obligate CO” resulting in genome instability.

### DEHP-induced chromosome morphology defects in late diakinesis may contribute to impaired embryogenesis and increased chromosome non-disjunction

The presence of chromosomal aggregates and aberrantly condensed bivalents in -1 oocytes at late diakinesis suggest the presence of unrepaired DNA damage bypassing both HR-mediated repair and culling by the apoptotic machinery earlier during pachytene. It has been shown that nuclei carrying unrepaired chromosomes upon exit from pachytene can engage in alternate error-prone repair pathways, such as intersister recombination or non-homologous end joining (NHEJ), often resulting in chromosomal aggregates suggestive of chromosome end-to-end fusions [31, 49, 65]. Furthermore, bivalents exhibiting condensation defects may suggest problems with the condensin machinery, which drives rapid chromosome decondensation and re-condensation during late prophase I remodeling following CO formation [66]. Alternatively, chromosome decondensation may be affected by impaired AKIRIN/AKIR-1 activity, previously shown to mediate chromosome condensation in a condensin-independent manner [67]. Given that we did not observe univalents in diakinesis oocytes despite the detection of CO-deficient chromosomes in DSB-1 lagging pachytene nuclei, it is possible that cohesin-independent and/or recombination-independent linkages, shown to be resolved by condensin, may preclude the premature dissociation of CO-deficient homologs before the completion of meiosis I [66]. On the other hand, molecular events affecting the expression/regulation/stability of cohesin complex components at the gene or protein level may compromise LAB-1 and AIR-2 bivalent association in late diakinesis, as the association of these proteins to chromosomes is unaffected at earlier stages of chromosome remodeling. Alternatively, defects impinging on HTP-1 and HTP-2 expression or function may preclude maintenance of LAB-1 chromosomal association [68, 69]. In either case, cohesin-independent linkages could maintain sister chromatid cohesion despite the absence of LAB-1.

Analysis of the first embryonic division revealed a higher incidence of chromosome congression failure at metaphase and chromatin bridges at anaphase in DEHP compared to DMSO-exposed worms. While a direct impact of DEHP on mitosis is likely, we cannot rule out the possibility that the defects observed during embryogenesis may have been carried over from meiosis. While DEHP-induced congression failure has not been previously described, several studies suggest that congression failure may stem from perturbations to spindle machinery components such as microtubules, actin filaments and the dynein and kinesin motor proteins or to problems with the interactions between kinetochores and the spindle. DEHP was recently shown to disrupt meiotic spindle assembly and chromosome alignment by reducing cytoskeletal actin expression in mice [70, 71]. In depletion studies of human kinesins, involved in the regulation of the attachment of spindle MTs to kinetochores, abnormal congression and chromosome misalignment was observed [71]. Finally, in *C*. *elegans*, defects in plus-end directed forces mediated by the chromokinesin KLP-19 and the ring complex also resulted in failure of chromosomes to congress [72]. Alternatively, chromosome congression defects may be due to problems in chromosome condensation given that the compromised function of the centromeric histone variant CeCENPA or the condensin II complex component HCP-6 result in chromosome condensation and congression defects in *C*. *elegans* embryos [66, 73]. In addition, both the defects we observed during meiotic prophase I and the observed mitotic defects may account for the mild increase in the incidence of males and embryonic lethality detected both by plate phenotyping and our recent HT chemical screen [33]. The low levels, however, may be explained in part by mechanisms set in place during meiotic divisions to recognize and eliminate aberrant chromosomes *via* polar body extrusion thus protecting the chromosomal integrity of the developing embryo [74].

### Elevated DSB and CO designation levels as well as changes in gene expression may be linked to DEHP-induced alterations in chromosome structure

The interplay between chromatin modifications and the lengths of both chromatin loops and chromosome axes is central to the establishment of chromosome structure and, ultimately, to the regulation of gene expression. Analysis of meiotic condensin mutants in *C*. *elegans* showed that perturbations to chromosome structure influence the position and frequency of DSBs in the genome and, hence, of COs [51]. The elongated X chromosome SCs observed in a subset of DEHP-exposed nuclei suggest alterations in chromosome structure resulting in shorter chromatin loops and a more relaxed chromatin that could increase the DNA accessibility of DSB-promoting factors and increase DSB levels. This is further supported by our observation of increased levels of RAD-51 foci marking sites undergoing DSB repair and of two COSA-1 foci marking CO designated sites on X chromosomes with extended SC lengths in pachytene nuclei from DEHP-treated compared to DMSO-treated worms **(Fig 8)**. These observations dovetail with the reduced pSYP-4 signal observed in a subset of nuclei as in the absence of pSYP-4 the SC persists in a more dynamic state and chromosomes persist in a DSB formation permissive state [53]. Moreover, these changes in chromosome structure may account for the statistically significant differences observed in the frequency of nuclei with different levels of COSA-1 foci between the two chemically-treated groups **(S4 Fig)** as it was previously shown that extension of the X axis length, upon disruption of any condensin subunit I, alters CO number and distribution [51]. Alternatively, the induction of a greater number of DSBs may be attributed to the interaction of DEHP or its metabolites with the peroxisome proliferator-activated receptors (PPARs), a nuclear family of receptors that function as transcription factors that activate genes involved in steroidogenesis and antioxidative stress [75, 76]. Given that stimulation of PPAR by DEHP or its metabolites activates an oxidative stress response, leading to DNA fragmentation and increased apoptosis of either sperm or oocytes in multiple organisms, we cannot discard the possibility that an excess number of DNA lesions may result from oxidative stress damage mediated by the interaction between DEHP and the *C*. *elegans* PPAR homolog NHR-49 [34, 70, 77, 78]. Several studies have shown that elevated exposure to DEHP in humans was associated with 8-hydroxy-deoxyguanosine, a biomarker of oxidative damage to DNA [79–81].

PRMT-5 has been implicated in the negative regulation of DNA damage-induced apoptosis in mammals and worms. While in mammals PRMT-5 can directly modify p53, in *C*. *elegans* the interaction between PRMT-5 and CBP-1, a co-factor of CEP-1, promotes the CEP-1-dependent transcriptional activity of *egl-1* thus exerting a regulatory control over apoptosis [82]. Therefore, the elevated levels of p53/CEP-1-dependent apoptosis may be associated with downregulation of *prmt-5* in DEHP-exposed gonads. Downregulation of genes exerting transcriptional control over pro-apoptotic factors was previously shown for bovine oocytes exposed to both DEHP and MEHP [83–85]. Furthermore, *prmt-5* downregulation may be partly accountable for the excess unrepaired DNA damage observed in DEHP-exposed germlines as depletion or inhibition of PRMT5 in mouse hematopoietic cells induces aberrant RNA splicing of the epigenetic regulator and DNA repair factor TIP60 (KAT5), a histone H4 acetyl-transferase, leading to impaired HR DNA repair while favoring repair via the NHEJ repair pathway [86]. Finally, a potential anti-estrogenic activity of DEHP, through its activation of estrogen receptors (ERs), ERα and ERβ, may account for the observed change in *prmt-5* expression. In mice, DEHP can impair estrogen synthesis and modulate the methylation of the meiotic-specific *STRA8* gene through estrogen receptors [87]. In zebrafish, low doses of DEHP were shown to reduce the mRNA levels of hormones controlling follicle development, oocyte maturation and ovulation, including the luteinizing hormone (LH) [88].

Taken together, our findings provide new insights into how environmentally-relevant levels of DEHP impair meiosis by linking two non-mutually exclusive processes to increased DSB levels: an impaired negative feedback loop normally set in place to downregulate DSB formation and changes in chromosome structure which may increase chromatin accessibility to DSB-promoting factors. Finally, coupled to the wide body of research on DEHP reprotoxicity, our results highlight a need for either reducing the use of DEHP as a mainstream plasticizer or identifying and advocating for the use of alternate and safer substitutes with the goal of minimizing the risk of human exposure.

## Materials and methods

### Strains

*C*. *elegans* lines were cultured at 20°C as described in [89], except for the *glp-1(bn18)(I);col-121 (nx3)(IV)* mutant which was cultured at 15°C. All lines used in these analyses carry *col-121(nx3)(IV)*, which increases cuticle permeability to chemicals allowing for low-dose studies [35]. The following strains were used in this study: *Pxol-1*::*GFP(V); col-121(nx3)(IV)*, *cep-1(Ig12501)(I*); *col-121(nx3)(IV)*, *rad-54(ok615)(I)/ht2(I;III); col-121(nx3)(IV)*, *glp-1(bn18)(I); col-121(nx3)(IV)*, *Ppie-1*::*GFP*::*COSA-1(I); col-121(nx3)(IV)*, and *H2B*::*mCherry; γ-tubulin*::*GFP; col-121(nx3)(IV)*.

### Chemicals

Dimethylsulfoxide (DMSO, ≥99.9%) and diethylhexyl phthalate (Pestanal, ≥98%) were purchased from Millipore-Sigma (Burlington, MA).

### Plate phenotyping analysis

Worms were continuously exposed to chemicals from eggs until adulthood (specifically, until 24 hours post the L4 larval stage). Embryos were collected from gravid adults treated with potassium hypochlorite solution as described in [90]. Washed embryos were plated onto cholesterol-free Nematode Growth Medium (NGM) agar plates (seeded with OP50 bacteria) containing final concentrations of DMSO (0.1%) and DEHP (10 μM, 100 μM and 500 μM). Chemical-containing plates were always prepared two to three days prior to exposure. Following 2.5 days of incubation at room temperature (RT), 10 to 22 L4-stage larvae were singled onto DMSO- or DEHP-containing cholesterol-free NGM agar plates and transferred onto chemical-containing plates every 24 hours for four consecutive days at RT. Brood size, % embryonic viability, % larval lethality and % males were determined by scoring the total number of eggs laid, the total number of eggs hatched, the total number of worms dying as larvae and the total number of males from among the total number of adult progeny, respectively.

### DAPI analysis of whole worm germlines

Carnoy’s fixation of >20 whole worms was performed 24 hours post-L4 per chemical treatment. Fixed worms were re-hydrated with M9 medium and stained with DAPI-containing Vectashield from Vector Laboratories (Burlingame, CA). Images of defects in chromosome morphology were visualized using a fluorescence Leica DM5000B microscope.

### Quantitative germline apoptosis analysis

Chemical exposures (10 μM, 50 μM, 100 μM, 250 μM and 500 μM) were done as described above. Apoptotic germ cell corpses were scored in the germlines of adult hermaphrodites, analyzed 24 to 26 hours post-L4 stage, following incubation with Acridine Orange (AO) for 2 hours at RT [91]. Apoptotic germ cell corpses from a minimum of 27 worms were visualized using a Leica DM5000B fluorescence microscope. Statistical analysis was done using an unpaired two-tailed Mann-Whitney test with 95% C.I.

### Germline immunostainings

For germline immunostainings, whole-mount gonads (collected 24 hours post-L4 stage) were dissected, fixed with 1% or 4% paraformaldehyde and immunostained as in [92].

Primary antibodies were used at the following final dilutions: rabbit α-RAD-51 (1:10,000; Novus Biological (SDI)), guinea pig α-SUN-1 Ser8-pi (1:700;[42]), guinea pig α-DSB-1 (1:500; [52]), rabbit α-LAB-1 (1:200; [47]), phosphohistone H3 (1:2,000; Cell Signaling), rabbit α-PLK-2 (1:100; [93]), goat α-SYP-1 (1:3,000; [94]), rabbit α-HIM-8 (1:500; SDI), guinea pig α-HIM-8 (1:100); [57]), rabbit α-phosphorylated SYP-4 S69 (1:100; [53]), and chicken α-GFP (1:500; Abcam). Secondary antibodies were purchased from Jackson ImmunoResearch Laboratories (West Grove, PA) and used at the following dilutions: α-rabbit Cy-3 (1:200), α-goat Cy-3 (1:200), α-guinea pig Cy-5 (1:100), α-rabbit Cy-5 (1:100), α-chicken Alexa 488 (1:500). α-mouse Alexa 488 (1:300) and α-rabbit Alexa 488 (1:500) were purchased from Cell Signaling. Immunofluorescence imaging was performed using a Delta Vision system equipped with an IX-70 microscope (Olympus, Waltham, MA) and a cooled CCD camera (Applied Precision, Pittsburg, PA). Images were collected using 60X and 100X objectives and Z-stacks were set at 0.2 μm thickness intervals. Image deconvolution was done using the SoftWoRX 3.3.6 software (Applied Precision).

### Time course RAD-51 quantification analysis

RAD-51 immunostained gonads were divided into seven zones and levels of RAD-51 foci on nuclei for each zone was quantified as described in [92]. Five germlines were scored *per* chemical treatment from three independent biological replicates. The average number of nuclei scored *per* zone for each chemical was as follows: DMSO: zone 1 = 131, zone 2 = 163, zone 3 = 131, zone 4 = 134, zone 5 = 130, zone = 114, and zone 7 = 93. DEHP: zone 1 = 144, zone 2 = 168, zone 3 = 140, zone 4 = 127, zone 5 = 126, zone 6 = 104, and zone 7 = 92.

For the RAD-51 time course analysis in a *rad-54; col-121(nx3)* background three and four germlines were scored for DMSO and DEHP, respectively, from three independent repeats. The average number of nuclei scored per zone for each chemical was as follows: DMSO: zone 1 = 62, zone 2 = 90, zone 3 = 90, zone 4 = 72, zone 5 = 46, zone 6 = 39, and zone 7 = 39. DEHP: zone 1 = 104, zone 2 = 132, zone 3 = 101, zone 4 = 86, zone 5 = 81, zone 6 = 68, and zone 7 = 43.

Data was plotted in GraphPad Prism and subjected to statistical analysis using an unpaired two-tailed Mann-Whitney test with 95% C.I.

### Quantification of SC length, RAD-51 foci and GFP::COSA-1 foci on the X chromosome

Whole mounted gonads from 24 hours post-L4 worms were co-immunostained for SYP-1 (central region component of the SC), HIM-8 (pairing center end protein that marks one end of the X chromosome) and either RAD-51 (DSB repair site marker) or anti-GFP to recognize GFP::COSA-1 (CO designation marker). X chromosomes marked by HIM-8 in pachytene nuclei were traced in 3D along the SYP-1 signal and straightened computationally using Priism 4.7 for measuring SC lengths and quantification of either RAD-51 or COSA-1 foci along the X chromosome as previously described [95]. Data was collected from seven independent biological repeats. A total of 176 nuclei for DMSO and 197 nuclei for DEHP from 53 and 61 gonads, respectively, were scored for SC length measurements. A total of 47 nuclei (DMSO) and 60 nuclei (DEHP) were scored for RAD-51 from 13 and 17 gonads, respectively, while 55 nuclei (DMSO) and 60 nuclei (DEHP) were scored for GFP::COSA-1 foci from 19 and 23 gonads, respectively.

### Quantification of GFP::COSA-1 foci in late pachytene nuclei

Quantification was performed by scoring the number of GFP::COSA-1 foci in nuclei in the last six rows of pachytene. Quantification on DSB-1 laggers was restricted to late pachytene as nuclei in this region have six GFP::COSA-1 foci in wild type. The mean number of GFP::COSA-1 foci was 5.9 as determined by scoring DMSO (n = 485) and DEHP (n = 461) late pachytene nuclei (17 gonads were scored from three biological repeats); n = number of nuclei scored. Deconvolved full nuclei projection images were used for this analysis. Statistical analysis was done using an unpaired two-tailed Mann-Whitney test with 95% C.I. A χ^2^ test was applied to assess variability in the distribution of GFP::COSA-1 foci.

### Live imaging of first embryonic division

24 to 30 hours post-L4 *H2B*::*mCherry; γ-tubulin*::*GFP; col-121(nx3)(IV)* exposed adult hermaphrodites were mounted on 3% agarose pads and immobilized with 0.01% Levamisol (final concentration) in 1X EGG buffer. Images of the first embryonic division were collected at 10 second intervals for 5 minutes with a 60X objective on an IX-70 microscope (Olympus, Waltham, MA) and a cooled CCD camera (CH350; Roper Scientific) controlled by the DeltaVision system (Applied Precision, Pittsburg, PA).

### Quantitative RT-PCR analysis

The temperature sensitive (ts) *glp-1(bn18)(I); col-121(nx3)(IV)* mutant background was used for qRT-PCR analysis. Worms were maintained and exposed to chemicals at 15°C and 15 hours post-exposure 100 L1-stage larvae were shifted to 25°C to block gonad development. Approximately 30 to 50 worms were collected 24 hours post-L4 stage and transferred to 100 μl Trizol. RNA was extracted as described by the manufacturer’s instructions. cDNA was produced by subjecting RNA to reverse transcription using iSCRipt (Biorad) and quantitative real time PCR was performed using the SsoFast EvaGreen supermix (Biorad) according to the manufacturer’s instructions. Experiments were done in three technical repeats from four biological runs. Cq numbers were normalized to *gpd-1* and statistical analysis was done by comparing DEHP-treated samples with the normalized values of vehicle control (DMSO)-treated samples. Statistical analysis was done using an unpaired two-tailed *t*-test.

### Mass spectrometric analysis of DEHP and its metabolites

Internal concentrations of DEHP and five of its metabolites in worm lysates were qualified. Extraction and analysis were performed by using the protocol similar to that reported previously [33]. Briefly, DEHP was extracted with hexane and analyzed by gas chromatography mass spectrometry (GC-MS). For the measurement of DEHP metabolites [five metabolites: mono-2-ethylhexyl phthalate (mEHP), Mono-(2-ethyl-5-carboxypentyl) phthalate (mECPP), mono-(2-ethyl-5-hydroxyhexyl) phthalate (mEHHP), mono-(2-ethyl-5-oxohexyl) phthalate (mEOHP) and mono-[(2-carboxymethyl) hexyl] phthalate, (mCMHP)] in *C*. *elegans*, we adopted the procedure that was used previously for mono-butyl phthalate (mBP) measurement [33]. Briefly, DEHP metabolites were extracted using solid-phase extraction (SPE) after enzymatic deconjugation and analyzed using high performance liquid chromatography-tandem mass spectrometry (HPLC-MS/MS). Quality control samples include analysis of National Institute of Standards and Technology (NIST) standard reference materials (SRM 3672 and SRM 3673) for DEHP metabolites in urine. The recoveries of DEHP metabolites from SRM ranged from 78 to 125%. There were trace levels of DEHP metabolites found in procedural blanks (mEHP: 1.2 ng/mL, mECPP: 0.30 ng/mL and mEHHP: 0.20 ng/mL) and these values were subtracted for those in worm samples to report final concentrations.

## Supporting information

S1 Fig Synaptonemal complex (SC) formation is not altered by DEHP exposure. High-resolution images of pachytene nuclei immunostained with SYP-1 (green) and co-stained with DAPI (blue) show continuous tracks for this central region component of the SC at the interphase between DAPI-stained chromosomes (homologs) in a manner indistinguishable from vehicle alone (0.1% DMSO) exposed worms. Between five to six gonads from two biological repeats were scored for DMSO (n = 130) and DEHP (n = 157); n = number of nuclei scored. Scale bar, 3 μm.
(TIF)

S2 Fig Frequency distributions of DSB-1 laggers in chemically-exposed germlines. A comparison of the frequency distribution of DSB-1 laggers in DMSO- and DEHP-exposed germlines shows an increasing rightward shift (x-axis) in the number of laggers in DEHP-exposed germlines relative to the DMSO control.
(TIF)

S3 Fig DEHP-exposed germlines displayed reduced PLK-2 signal. (**A**) High-resolution images of whole mounted gonads of chemically-exposed worms immunostained against PLK-2 and DSB-1. Scale bar, 5 μm. (**B**) High-resolution images of mid-pachytene DSB-1 laggers displaying reduced PLK-2 signal. Scale bar, 3 μm.
(TIF)

S4 Fig Analysis of COSA-1::GFP levels in chemically-exposed worms. **(A)** High-resolution images of nuclei in the last six rows of late pachytene stained with anti-GFP (green) and co-stained with DAPI (blue). Scale bar, 5 μm. (**B**) Quantification of the number of GFP::COSA-1 foci in the last six rows of late pachytene when six GFP::COSA-1 foci are detected per nucleus in wild type representing six COs (one per homolog pair). The number of nuclei scored for DMSO and DEHP were n = 485 and n = 461, respectively (from 17 gonads each from three biological repeats). (**C**) Graph showing the distribution in the number of late pachytene nuclei (y-axis) containing different numbers of GFP::COSA-1 foci (0 through 10) (x-axis) for each chemical treatment. χ^2^ = 19.9, *P = 0.0029.
(TIF)

S1 Table Raw data set. Raw data for dose-response curves, plate phenotyping, SUN-1 (pS8) laggers count, bivalent morphology analysis, LAB-1 and phosphohistone H3 (pS10) localization analysis, live imaging count, germ cell apoptosis count, RAD-51 foci count, DSB-1 laggers and GFP::COSA-1 counts, qRT-PCR analysis and X chromosome SC lengths, RAD-51 and GFP::COSA-1 counts.
(XLSX)
