# Supplementary figures and images for: Environmentally-relevant exposure to diethylhexyl phthalate (DEHP) alters regulation of double-strand break formation and crossover designation leading to germline dysfunction in Caenorhabditis elegans
Source: PLoS Genet. 2020 Jan 9;16(1):e1008529. doi: 10.1371/journal.pgen.1008529 (PMC6952080; doi:10.1371/journal.pgen.1008529)

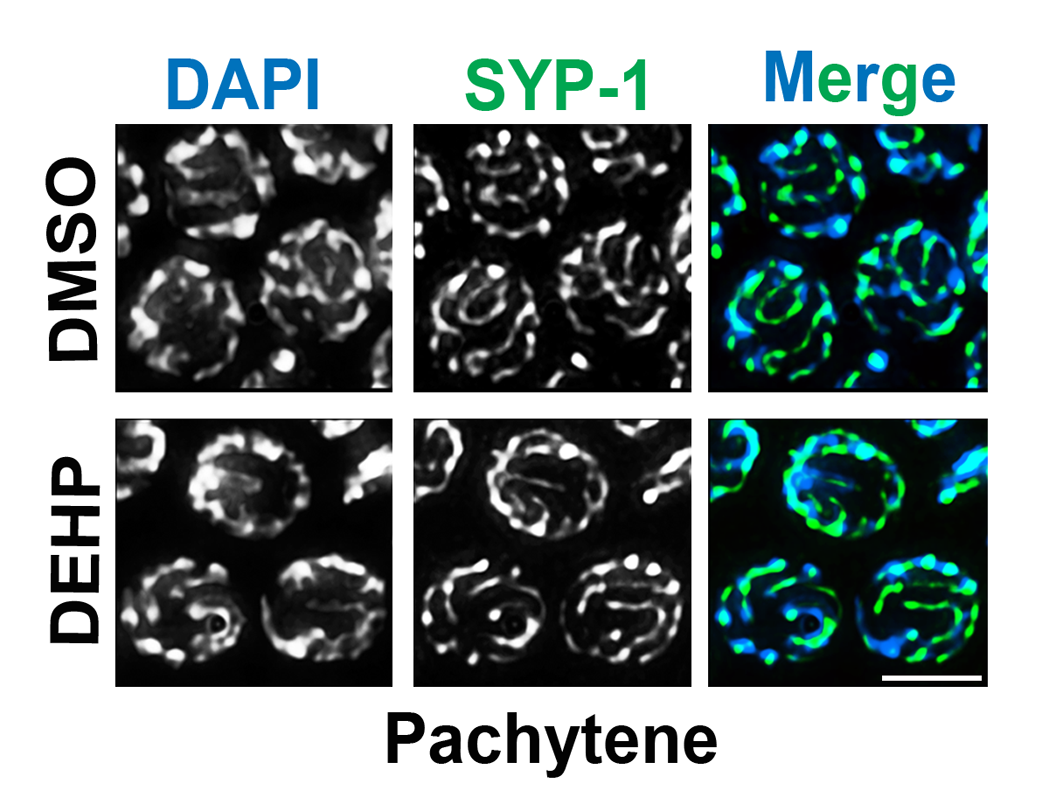

Supplement: S1 Fig — High-resolution images of pachytene nuclei immunostained with SYP-1 (green) and co-stained with DAPI (blue) show continuous tracks for this central region component of the SC at the interphase between DAPI-stained chromosomes (homologs) in a manner indistinguishable from vehicle alone (0.1% DMSO) exposed worms. Between five to six gonads from two biological repeats were scored for DMSO (n = 130) and DEHP (n = 157); n = number of nuclei scored. Scale bar, 3 μm. (TIF) [file pgen.1008529.s001.tif]

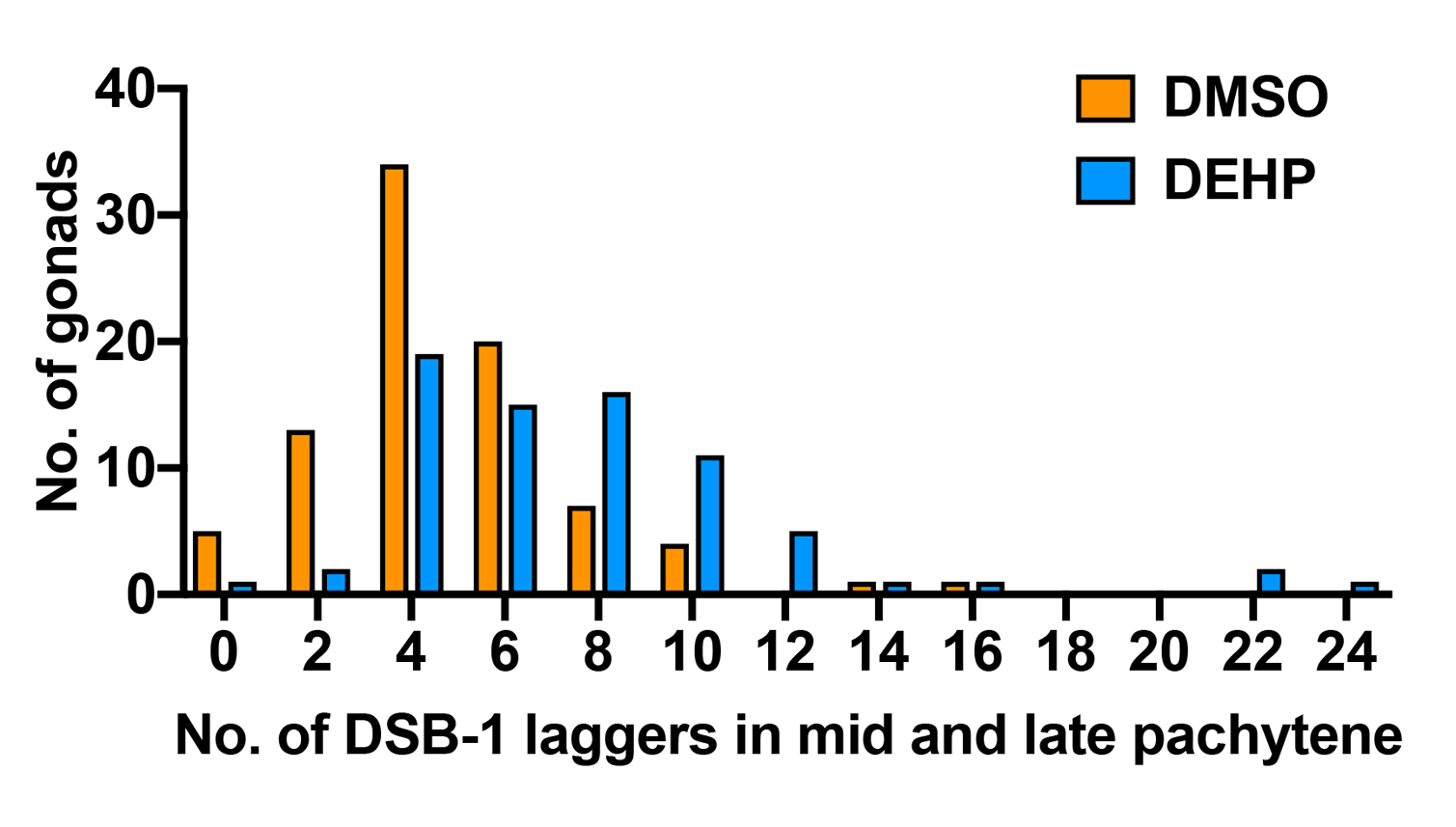

Supplement: S2 Fig — A comparison of the frequency distribution of DSB-1 laggers in DMSO- and DEHP-exposed germlines shows an increasing rightward shift (x-axis) in the number of laggers in DEHP-exposed germlines relative to the DMSO control. (TIF) [file pgen.1008529.s002.tif]

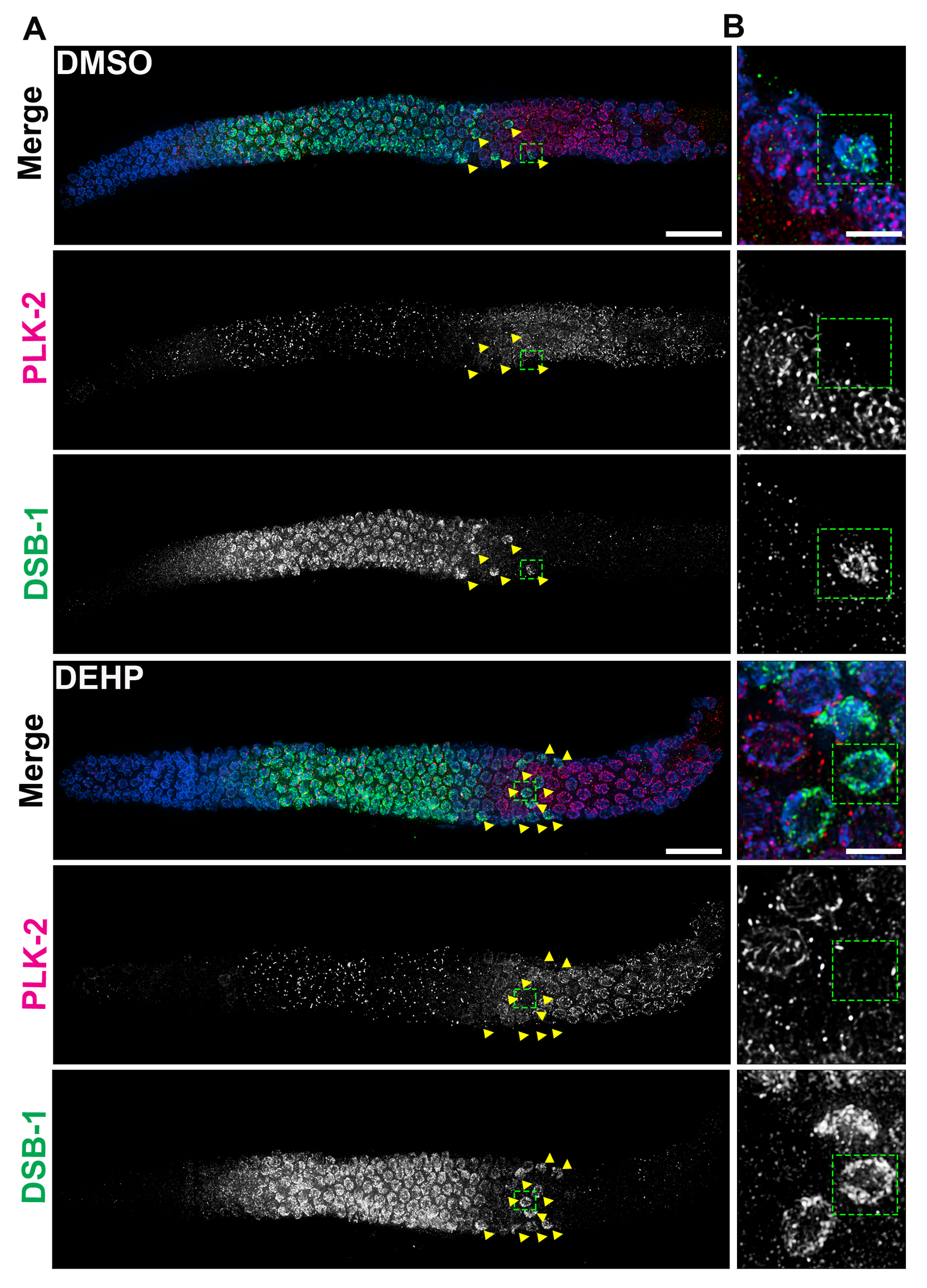

Supplement: S3 Fig — (A) High-resolution images of whole mounted gonads of chemically-exposed worms immunostained against PLK-2 and DSB-1. Scale bar, 5 μm. (B) High-resolution images of mid-pachytene DSB-1 laggers displaying reduced PLK-2 signal. Scale bar, 3 μm. (TIF) [file pgen.1008529.s003.tif]

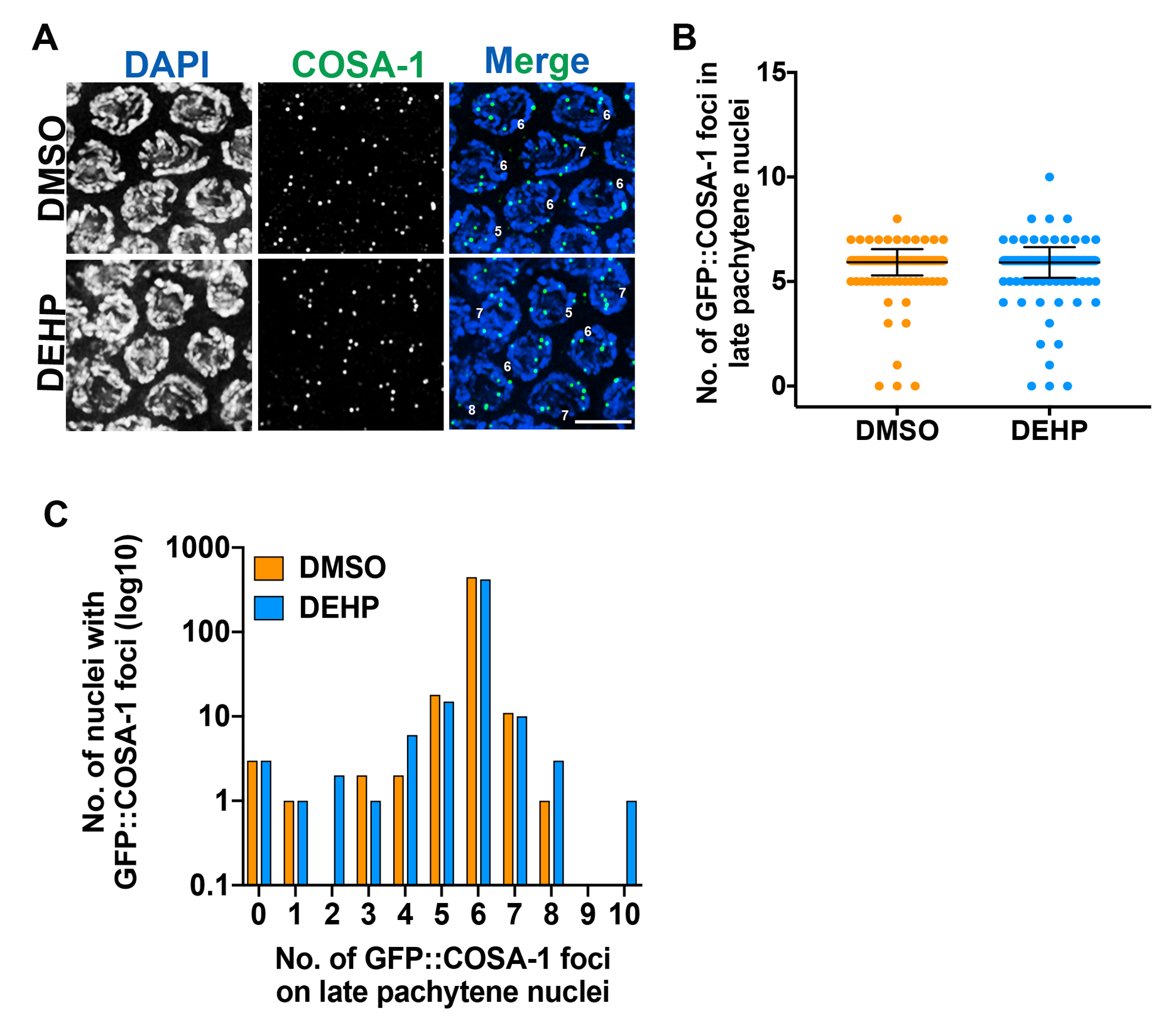

Supplement: S4 Fig — (A) High-resolution images of nuclei in the last six rows of late pachytene stained with anti-GFP (green) and co-stained with DAPI (blue). Scale bar, 5 μm. (B) Quantification of the number of GFP::COSA-1 foci in the last six rows of late pachytene when six GFP::COSA-1 foci are detected per nucleus in wild type representing six COs (one per homolog pair). The number of nuclei scored for DMSO and DEHP were n = 485 and n = 461, respectively (from 17 gonads each from three biological repeats). (C) Graph showing the distribution in the number of late pachytene nuclei (y-axis) containing different numbers of GFP::COSA-1 foci (0 through 10) (x-axis) for each chemical treatment. χ2 = 19.9, *P = 0.0029. (TIF) [file pgen.1008529.s004.tif]
